# BuD, a helix–loop–helix DNA-binding domain for genome modification

**DOI:** 10.1107/S1399004714011183

**Published:** 2014-06-29

**Authors:** Stefano Stella, Rafael Molina, Blanca López-Méndez, Alexandre Juillerat, Claudia Bertonati, Fayza Daboussi, Ramon Campos-Olivas, Phillippe Duchateau, Guillermo Montoya

**Affiliations:** a Macromolecular Crystallography Group, Structural Biology and Biocomputing Programme, Spanish National Cancer Research Centre (CNIO), Calle de Melchor Fernández Almagro 3, 28029 Madrid, Spain; b Structural Biology Group, Novo Nordisk Foundation Center for Protein Research, Faculty of Health and Medical Sciences, University of Copenhagen, Blegdamsvej 3B, 2200 Copenhagen, Denmark; c Spectroscopy and NMR Unit, Spanish National Cancer Research Centre (CNIO), Calle de Melchor Fernández Almagro 3, 28029 Madrid, Spain; d Cellectis, 8 Rue de la Croix Jarry, 75013 Paris, France

**Keywords:** gene targeting, protein–DNA interaction, genetics

## Abstract

DNA editing offers new possibilities in synthetic biology and biomedicine for modulation or modification of cellular functions to organisms. However, inaccuracy in this process may lead to genome damage. To address this important problem, a strategy allowing specific gene modification has been achieved through the addition, removal or exchange of DNA sequences using customized proteins and the endogenous DNA-repair machinery. Therefore, the engineering of specific protein–DNA interactions in protein scaffolds is key to providing ‘toolkits’ for precise genome modification or regulation of gene expression. In a search for putative DNA-binding domains, BurrH, a protein that recognizes a 19 bp DNA target, was identified. Here, its apo and DNA-bound crystal structures are reported, revealing a central region containing 19 repeats of a helix–loop–helix modular domain (BurrH domain; BuD), which identifies the DNA target by a single residue-to-nucleotide code, thus facilitating its redesign for gene targeting. New DNA-binding specificities have been engineered in this template, showing that BuD-derived nucleases (BuDNs) induce high levels of gene targeting in a locus of the human haemoglobin β (HBB) gene close to mutations responsible for sickle-cell anaemia. Hence, the unique combination of high efficiency and specificity of the BuD arrays can push forward diverse genome-modification approaches for cell or organism redesign, opening new avenues for gene editing.

## Abstract 2

Crystal structures of BurrH and the BurrH–DNA complex are reported.

## 1. Introduction   

The tailoring of homing endonucleases (HEs; Redondo *et al.*, 2008 ▶; Muñoz *et al.*, 2011 ▶) and other custom-made proteins, such as zinc fingers (ZFs; Urnov *et al.*, 2010 ▶), transcription activator-like effector domains (TALEs; Miller *et al.*, 2011 ▶) and the recently introduced CRISPR/Cas systems (Cong *et al.*, 2013 ▶; Mali *et al.*, 2013 ▶), has demonstrated the potential of this approach to create new specific instruments to target genes for activation, repression or repair (Prieto *et al.*, 2012 ▶).

These tools will be particularly important in organism design and medical applications, where they can be applied as *ex vivo* therapies in human monogenic diseases (Redondo *et al.*, 2008 ▶). The constant release of new genomic data from diverse organisms allows the identification of novel DNA-binding proteins that could improve the current repertoire. We identified BurrH in *Burkholderia rhizoxinica*, a symbiotic bacterium found in the cytosol of *Rhizopus* microspores (Juillerat *et al.*, 2014 ▶). We expressed, purified and solved the structure of this protein, which is able to specifically recognize its DNA target. The biophysical and structural analysis permitted the design of a new class of specific nucleases, demonstrating the potential of this protein template to perform efficient and specific genome editing in human cells.

**Figure 1:**
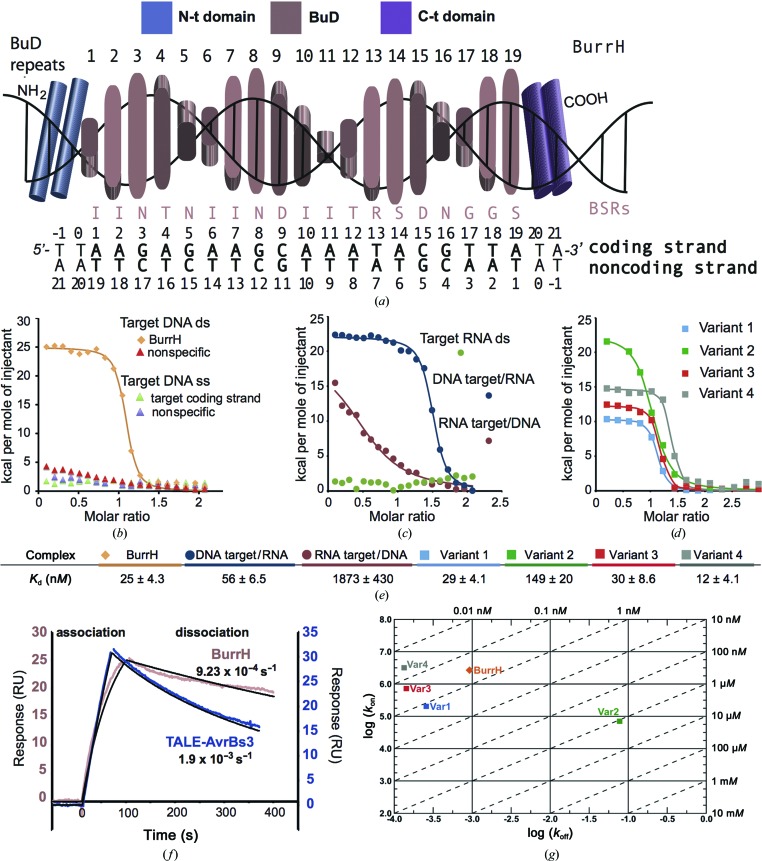
BurrH recognizes its target DNA with high affinity and specificity in an endothermic reaction. (*a*) Scheme of the BurrH domain structure. The central DNA-binding domain contains the BuD repeats with the residues involved in DNA recognition (BSRs; Supplementary Figs. S1 and S2). The sequence of the coding (the strand defined by the single amino acid-to-nucleotide correspondence) and noncoding (the complement of the coding strand) strands of the oligonucleotide used in the biophysical characterization and crystallization is depicted below. The BurrH target sequence is shown in bold. (*b*) ITC binding curves of BurrH. The protein specifically recognizes its double-strand (ds) DNA target. BurrH is not able to bind DNA duplexes with other sequences or single-strand (ss) DNAs containing its target sequence. (*c*) ITC binding curves of BurrH using DNA-RNA hybrids and RNA duplexes as targets. (*d*) ITC binding curves of BurrH-based variants display the same thermodynamic behaviour as the wild-type protein (see Supporting Information and Supplementary Fig. S4). (*e*) Table summarizing the *K*_d_ values of the ITC analysis. The affinities of the redesigned variants are similar to the wild-type protein except for Var2. (*f*) SPR analysis of BurrH target binding compared with AvrBs3 TALE. The BuD array presents a fast association and low dissociation behaviour (see Supplementary Fig. S5). In both cases 12.5 n*M* protein was flowed over the chip for 95 s. Mono exponential fits are shown in black for the curves (Supplementary Fig. S5). (*g*) On–off rate map showing the values of the association and dissociation rate constants and the resulting affinity as obtained from SPR. Dashed diagonals represent different *K*_d_ values (indicated on the upper and right axes). Positions along the same diagonal have the same *K*_d_ values but different *k*_on_ and *k*_off_ values.

BurrH comprises 794 residues and three different regions (Fig. 1 ▶ *a*, Supplementary Fig. S1^1^). The central section contains 19 repeats of a new modular domain (BurrH domain; BuD), while the N- and C-terminal regions include two degenerate BuD-like repeats (Fig. 1 ▶ *a*). Each BuD repeat is composed of 33 residues, only eight of which are strictly conserved between the repeats (Supplementary Figs. S1 and S2).

## 2. Materials and methods   

### 2.1. Expression, purification and crystallization   

Protein expression, purification, protein–DNA complex formation and crystallization have been described in Stella *et al.* (2014 ▶).

### 2.2. Fluorescence anisotropy   

The dissociation (*K*_d_) constants between BurrH and its target DNA were estimated from the change in fluorescent polarization upon protein addition using oligonucleotides labelled with 6-FAM at the 5′-end. The optimal concentration of the 6-FAM-DNAs was determined empirically by measuring the fluorescence polarization of serially diluted 6-FAM-labelled DNA samples (Molina *et al.*, 2012 ▶). The concentration of the 6-FAM-labelled DNAs ranged between 20 and 40 n*M* and that of the BurrH protein was increased up to 1000 n*M*. Both proteins and DNAs were dialyzed in buffer consisting of 25 m*M* HEPES pH 8, 150 m*M* NaCl, 0.2 m*M* TCEP. After incubation at 298 K for 10 min, the fluorescence polarization was measured in a black 96-well assay plate using a Wallac Victor 2V 1420 multilabel counter (PerkinElmer). The fitting of the data and the *K*_d_ calculations were performed as described previously (Molina *et al.*, 2012 ▶).

### 2.3. Isothermal titration calorimetry assays   

Isothermal titration calorimetry (ITC) experiments were conducted at 298 K on a MicroCal iTC200 instrument (MicroCal, GE Healthcare, UK). The buffer consisted of 25 m*M* HEPES pH 8, 150 m*M* NaCl, 0.2 m*M* TCEP. To ensure minimal buffer mismatch, protein and DNA samples were dialyzed against the same buffer. The syringe for the ligand contained DNA duplexes in a concentration range between 80 and 100 µ*M*. The thermostatic cell contained BurrH protein in a concentration range between 8 and 10 µ*M*. The corrected binding isotherms were fitted to a multiple but identical sites binding model using a nonlinear least-squares algorithm in the *Origin* 7.0 software (MicroCal) to obtain values of the equilibrium binding constant (*K*_a_), stoichiometry (*n*) and enthalpy changes (Δ*H*) and the *T*Δ*S* associated with DNA binding. The *K*_d_ was the inverse of the calculated *K*_a_ and the associated error was estimated using an error-propagation calculator (http://laffers.net/tools/error-propagation-calculator/).

### 2.4. Surface plasmon resonance   

Surface plasmon resonance experiments were performed on a Biacore X100 (GE Healthcare). A CM5 chip (GE Healthcare) was coated with streptavidin in order to be able to bind a biotinylated single-stranded oligonucleotide of 12 bases. This anchor was then used to attach the different double-stranded DNA fragments (containing an anchor-complementary overhang) examined in this study (Supplementary Fig. S5*a*). The duplex-containing DNA fragments were made just prior to use by mixing (in 10 m*M* Tris pH 8.0, 50 m*M* NaCl) at a final concentration of 0.5 m*M* a shorter oligonucleotide carrying the binding site of the proteins and a longer oligonucleotide complementary to both the shorter oligonucleotide and the anchor sequence in a 1.2:1 molar ratio. The mixture was heated to 368 K for 5 min followed by slow cooling to room temperature.

The CM5 chip was treated as follows. Firstly, streptavidin at 60 µg ml^−1^ in 10 m*M* sodium acetate buffer pH 4.5 was immobilized using amine-coupling chemistry and HBS-EP+ buffer [10 m*M* HEPES pH 7.4, 150 m*M* NaCl, 3 m*M* EDTA, 0.05%(*v*/*v*) surfactant P20] as running buffer. On average, 3000 response units (RUs) of streptavidin were immobilized on both flow cells. Secondly, the biotinylated oligonucleotide at 2.5 n*M* in running buffer was injected at a flow rate of 5 µl min^−1^ into both flow cells by consecutive manual pulses until 10 RUs were reached. Finally, the duplex DNA fragment diluted at 50–100 n*M* in 1 *M* NaCl was injected manually in short pulses at a 5 µl min^−1^ flow rate over flow cell 2 only (‘fc2’), leaving flow cell 1 (‘fc1’) as a control. Typically, 5–10 RUs of the target DNAs were immobilized for the kinetic analysis. The DNA fragments were removed from the anchor DNA on the CM5 chip by a series of pulses of 50 m*M* NaOH at 10 µl  min^−1^ until a stable baseline was observed. Therefore, the CM5 chip containing the biotinylated anchor could be reused with different DNA target sequences.

Affinity and kinetic experiments were carried out at flow rates of 10 and 30 µl min^−1^, respectively, at 298 K. Protein samples were prepared by serial dilutions (from 5 to 0.31 n*M* and from 2.5 to 0.125 µ*M* for targets with affinities in the low nanomolar and micromolar ranges, respectively) in HBS-EP+ running buffer starting from stocks of concentrated protein (200 µ*M*). Any protein that remained bound after a 3–6 min dissociation phase was removed by injecting regeneration buffer (0.05% SDS in HBS-EP+) for 12 s at 10 µl min^−1^, which regenerated the surface to the baseline value observed prior to protein injection. Measurements at each protein concentration were repeated at least twice. All responses were double-referenced. For kinetic analysis, data were globally fitted to a 1:1 interaction model with a correction for mass transport (as provided by the manufacturer’s software). For equilibrium analysis, the averaged response during the last 5 s before the injection stop was plotted against the protein concentration and fitted to a simple binding isotherm. All data processing and analysis were performed with the Biacore X100 Evaluation Software (version 2.0.1) from GE Healthcare.

### 2.5. Structure determination, model building and refinement   

The structure of BurrH in the apo form was determined by the single-wavelength anomalous diffraction (SAD) technique using a selenium derivative and a data set at the peak of the Se *K* absorption edge (λ = 0.98 Å). SAD data were collected from cooled crystals at 100 K using a PILATUS detector on the PXI-XS06 beamline at SLS Villigen, Switzerland. Data processing and scaling were accomplished by *XDS* (Kabsch, 2010 ▶). All methionines were substituted by selenomethionine and the 12 possible Se sites were identified using the *SHELX* package (Sheldrick, 2008 ▶). Initial phases were calculated at 2.45 Å resolution using the *AutoSolve* program included in *PHENIX* (Adams *et al.*, 2010 ▶). These initial phases were extended to 2.21 Å resolution using the same data set with the *PHENIX* *AutoBuild* routine. Native diffraction data sets (λ = 1.00 Å) were collected from cooled BurrH–DNA crystals at 100 K using a PILATUS detector on the PXI-XS06 (SLS Villigen, Switzerland) and XALOC beamlines (ALBA Synchrotron, Barcelona, Spain). The structure of the BurrH–DNA complex was determined by molecular replacement using *Phaser* (McCoy *et al.*, 2007 ▶) with a set of three BuD repeats selected from the apo BurrH structure as a search model. The initial model was remodelled manually with *Coot* (Emsley *et al.*, 2010 ▶) and refined using *PHENIX* (Adams *et al.*, 2010 ▶). Refinement and data-collection statistics are summarized in Table 1 ▶. The Ramachandran plot for the apo structure showed 99.73, 0.27 and 0% of the residues in the favoured, allowed and disallowed regions, respectively. The same plot for the protein–DNA structure exhibited 93.48, 6.13 and 0.40% of the residues in the favoured, allowed and disallowed regions, respectively. Identification and analysis of the protein–DNA hydrogen bonds and van der Waals contacts was performed with the *Protein Interfaces, Surfaces and Assemblies* service (*PISA*) at the European Bioinformatics Institute (http://www.ebi.ac.uk/msdsrv/prot_int/pistart.html).

### 2.6. Extrachromosomal single-strand assay (SSA) in yeast   

Scaffolds and DNA-targeting arrays were synthesized *de novo* (GeneCust) and subcloned in bacterial, yeast or mammalian (under the EF1α promoter) expression vectors. All yeast and mammalian expression constructs contained a nuclear localization sequence (NLS).

Nuclease-containing yeast strains (mutant) were gridded using a colony gridder (QPix II, Genetix) on nylon filters placed on solid agar containing YP-glycerol at ∼20 spots cm^−2^. A second layer, consisting of reporter-harbouring (target) yeast strains, was gridded on the same filter. The filters were incubated overnight at 303 K to allow mating and were then placed and incubated for 2 d at 303 K on medium lacking leucine (for the mutant) and tryptophan (for the target) with glucose (2%) as the carbon source to allow selection of diploids. To induce expression of the nuclease, the filters were transferred onto YP-galactose-rich medium for 48 h at 293, 298, 303 or 310 K. The filters were finally placed onto solid agarose medium containing 0.02% X-Gal in 0.5 *M* sodium phosphate buffer pH 7.0, 0.1% SDS, 6% dimethylformamide (DMF), 7 m*M* β-mercaptoethanol, 1% agarose and incubated at 310 K for up to 48 h to monitor nuclease activity through the β-galactosidase activity. The filters were scanned and each spot was quantified using the median values of the pixels constituting the spot. We attribute the arbitrary values 0 and 1 to white and dark pixels, respectively. β-Galactosidase activity is directly associated with the efficiency of homologous recombination and thus with the cleavage efficiency of the nuclease.

### 2.7. Nuclease transfection   

293H cells were cultured at 310 K with 5% CO_2_ in DMEM Complete medium supplemented with 2 m*M* l-glutamine, penicillin (100 IU ml^−1^), streptomycin (100 µg ml^−1^), amphotericin B (fongizone; 0.25 µg ml^−1^; Life Technologies) and 10% foetal bovine serum (FBS). Adherent 293H cells were seeded at 1.2 × 10^6^ cells in 10 cm Petri dishes 1 d before transfection. Cell transfection was performed using the Lipofectamine 2000 reagent according to the manufacturer’s instructions (Invitrogen). In brief, for targeted mutagenesis experiments, 2.5 µg of each of the two BurrH nuclease expression vector pairs and 50 ng GFP expression vector (5 µg final DNA) were mixed with 0.3 ml DMEM without FBS. After 5 min incubation, the DNA and Lipofectamine mixtures were combined and incubated for 25 min at room temperature. The mixture was transferred to a Petri dish containing the 293H cells in 9 ml Complete medium and then cultured at 310 K under 5% CO_2_. 3 d post-transfection, the cells were washed twice with phosphate-buffered saline (PBS), trypsinized and resuspended in 5 ml Complete medium, and the percentage of GFP positive cells was measured by flow cytometry (Guava EasyCyte) in order to monitor the transfection efficacy.

### 2.8. Targeted mutagenesis   

Cells were pelleted by centrifugation and genomic DNA was extracted using the DNeasy Blood & Tissue Kit (Qiagen) according to the manufacturer’s instructions. PCR of the endogenous locus was performed using locus-specific oligonucleotides and purified using the AMPure kit (Invitrogen). Amplicons were further analyzed by the T7 endonuclease assay as described previously (Valton *et al.*, 2012 ▶) or by deep sequencing using the 454 system (Roche).

### 2.9. Targeted gene insertion   

Cells were re-seeded 3 d post-transfection in three 96-well plates at a density of ten cells per well and cultured at 310 K for a further 15 d in DMEM Complete medium. The plasmidic donor DNA was composed of two homologous arms (959 and 1193 bp) separated by 29 bp of an exogenous sequence. The detection of targeted integration was monitored 18 d post-transfection by performing a locus-specific PCR amplification (Herculase II Fusion kit, Agilent). In these experiments, one primer was located within the heterologous insert of the donor DNA and the other on the genomic sequence outside of the homology arm (Supplementary Table S1). In addition, as we performed this experiment at ten cells per well, we had to take into account the transfection (as monitored by GFP positive cells) and plating (estimated to be of 30%) efficiencies to evaluate the TGI frequency (Daboussi *et al.*, 2012 ▶).

## 3. Results   

### 3.1. BurrH–DNA interaction   

The BuD repeats show 36% identity on average to those found in the AvrBs3 TALE (Juillerat *et al.*, 2014 ▶; Schornack *et al.*, 2013 ▶). Initially, the DNA sequence targeted by BurrH was predicted using the dipeptide code previously reported for TALEs (Boch *et al.*, 2009 ▶; Moscou & Bogdanove, 2009 ▶). However, new residues (Thr and Arg) at the 13th position of the repeat, which could potentially be involved in DNA recognition, suggested the presence of new interactions involved in determining protein–DNA specificity. Hence, we analyzed the nucleotide preference for these amino acids using a battery of oligonucleotides with all possible bases at these sites (Supplementary Fig. S3). Three of these duplexes showed affinities ranging from 30 to 40 n*M*. The DNA bearing A, A and T at positions 4, 12 and 13, respectively, displayed the highest affinity and was the only one that yielded crystals of the BurrH–DNA complex (Stella *et al.*, 2014 ▶); consequently, we performed the rest of the characterization using this target sequence.

Having assessed the base preferences of the residues involved in DNA recognition, we dissected the BurrH–DNA interaction. In contrast to other protein templates employed in genome editing [*i.e.* ZF (Deegan *et al.*, 2011 ▶), I-CreI (Molina *et al.*, 2012 ▶) and TALEs (Stella *et al.*, 2013 ▶)], which exhibit exothermic-driven reactions, isothermal titration calorimetry (ITC) revealed the endothermic entropy-driven nature of BurrH–DNA association (Fig. 1 ▶ *b*). This BuD is able to recognize its duplex DNA with high specificity and affinity (*K*_d_ = 25 n*M*), and it cannot bind the other tested duplexes with unrelated sequences or a single-strand DNA containing its target sequence (Fig. 1 ▶ *b*). Furthermore, BurrH does not recognize RNA duplexes and displays low affinity for a RNA-DNA hybrid containing its target sequence in the RNA (Fig. 1 ▶ *c*; Supplementary Fig. S4). However, BurrH can bind a DNA-RNA hybrid when this sequence is in the DNA strand, as reported for TALE (Yin *et al.*, 2012 ▶; Fig. 1 ▶ *c*, Supplementary Fig. S4). DNA-RNA hybrids are associated with different biological processes such as transcription and DNA replication, but also with infection by retroviruses. Thus, BurrH could offer opportunities to intervene in these processes. Other sequence preferences were introduced in BurrH, generating new variants (Fig. 1 ▶ *d*, Supplementary Fig. S4). These proteins were designed to bind sequences contained in the *CAPNS1* (calpain small subunit 1; variants 1 and 2) and *RAG1* (recombination activating gene 1; variants 3 and 4) human genes. Both the affinities and the balances between enthalpic and entropic contributions were similar to those of the wild-type protein, indicating that this new protein platform can be used to design new DNA specificities with minor binding interferences with other nucleic acids (Figs. 1 ▶ *b*–1 ▶ *e*, Supplementary Fig. S4).

The kinetic properties of BurrH–DNA interaction are crucial for evaluating its possible genome-modification applications. Surface plasmon resonance (SPR) was employed for this purpose. The target DNA was immobilized on a streptavidin chip (Supplementary Fig. S5*a*) and the BuD was assayed for binding (Supplementary Fig. S5*b*). Our data confirmed not only that BurrH exhibits a high affinity and specificity for its target but also significantly slower dissociation compared with the AvrBs3 TALE (Fig. 1 ▶ *f*). Moreover, BurrH does not display binding to other DNA duplexes, in contrast to TALE, which associates with BurrH target DNA (Supplementary Figs. S5*c* and S5*d*).

All of the engineered variants targeting different DNA sequences maintain similar thermodynamic characteristics (Supplementary Fig. S4*d*) and only variant 2, which exhibited the lowest affinity, displayed a higher off rate than BurrH (Fig. 1 ▶ *g*). The differences in the *K*_d_ values between ITC and SPR could arise from the different parameters that are used to quantify binding. Nevertheless, the differences observed are consistent and follow the same pattern in both cases. In summary, these tailored proteins displayed high specificity and did not show binding to any of the other duplexes tested (Supplementary Fig. S5*e*).

### 3.2. Crystal structures of BurrH and the BurrH–DNA complex   

**Figure 2:**
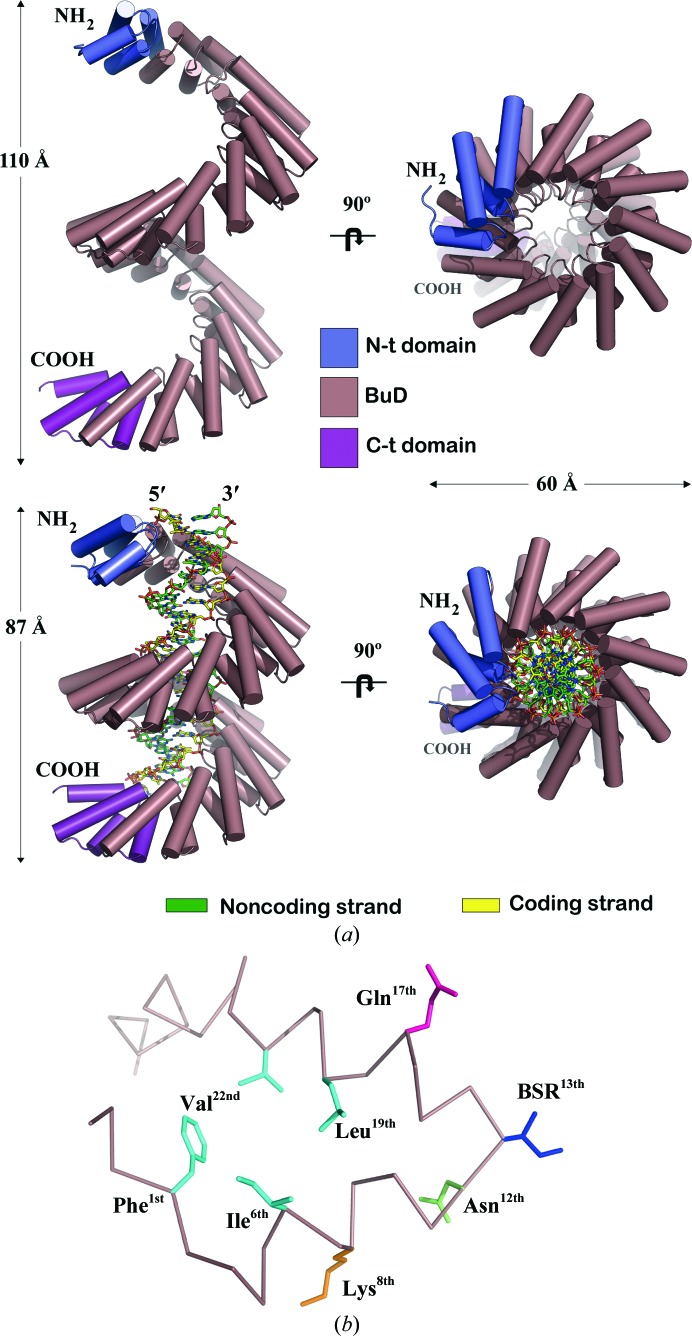
Crystal structures of BurrH and the BurrH–DNA complex. (*a*) Crystal structures of apo and DNA-bound BurrH (2.21 and 2.65 Å resolution, respectively). Cartoon representation of the crystal structures perpendicular to the longitudinal DNA axis (left panel) and along the DNA helix (right panel). The helical elements of BurrH are shown as cylinders and the duplex oligonucleotide is represented in stick mode. (*b*) Ribbon diagram of a BuD repeat. The side chains of the key residues (Supplementary Fig. S2) are shown in stick mode, including their positions in the repeat. Hydrophobic amino acids (Phe, Ile, Val and Leu) are coloured light blue, Gln magenta, Lys orange and the invariant Asn green.

To examine the molecular basis of the BurrH–DNA interaction, we crystallized and solved the apo and protein–DNA structures (see *Methods* [Sec sec2]; Fig. 2 ▶ *a*). The models were refined to 2.21 and 2.65 Å resolution, respectively (Table 1 ▶). BurrH resembles the solenoid protein families such as the tetratricopeptide (TPR; Scheufler *et al.*, 2000 ▶), pentatricopeptide (PPR; Yin *et al.*, 2013 ▶) and Sel1-like (SLR) repeat (Mittl & Schneider-Brachert, 2007 ▶) families. All of these families display α-helical elements with different degrees of conservation of their primary structure and superhelical topologies. Functionally, they are involved in protein–protein interactions and polynucleotide recognition. The crystal structures revealed the extensive conformational rearrangement of the protein after DNA recognition (Fig. 2 ▶ *a*, Supplementary Movie 1). BurrH shrinks 23 Å along the longitudinal axis wrapping the DNA molecule, which displays an almost unperturbed B-form. Upon DNA binding the protein is compressed like an accordion along the DNA, while the BuD bends spirally around the nucleic acid as shown in dHax3 (Deng *et al.*, 2012 ▶; Supplementary Movie 1). This compression is favoured by the presence of an inter-repeat hydrophobic patch built by some of the strictly conserved residues in the BuD repeats (Phe^1st^, Ile^6th^, Leu^19th^ and Val^22nd^ positions in the helix–loop–helix repeat; Figs. 2 ▶ *b* and 3 ▶ *a*, Supplementary Fig. S2). These amino acids located in strategic sites, together with the DNA contacts, promote the corkscrew shape of the DNA-bound complex (see Supporting Information).

**Figure 3:**
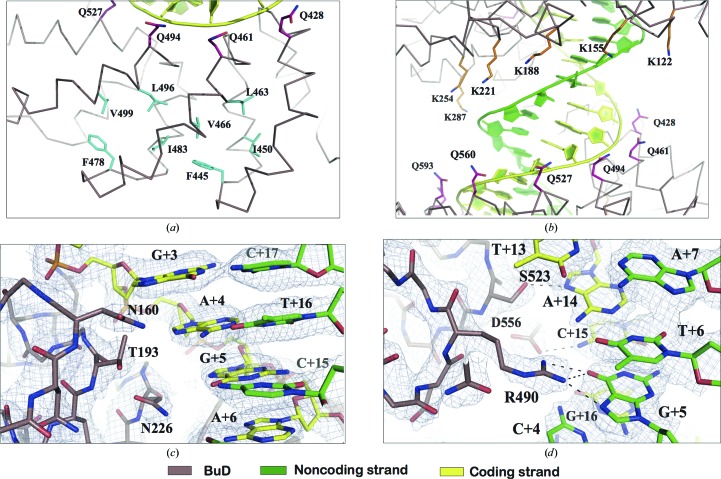
Detailed view of BurrH–DNA binding and the new BSR interactions. (*a*) Inter-repeat hydrophobic cluster built by four of the strictly conserved amino acids upon DNA binding. (*b*) General view of the protein–DNA association depicting the arrangement of the conserved polar stripes (composed of Lys/Arg and Gln at positions 8 and 17 of the BuD repeats, respectively) stabilizing the phosphate backbone of the noncoding and coding DNA strands. (*c*) Recognition of A_+4_ by Thr193 in the fourth BuD repeat. (*d*) Detailed view of the interaction of Arg490 with the duplex DNA establishing key interactions with both DNA stands. The electron-density map for all of the figures is a 2*F*_o_ − *F*_c_ σ_A_-weighted map contoured at 1.2σ.

The electrostatic potential of BurrH shows two electropositive stripes running along the protein which contact the phosphate backbones of the double helix (Supplementary Fig. S6). The coding strand interacts with one of these stripes composed of a conserved Gln at position 17 in the BuD repeats (2.5–3.5 Å distance from the phosphate backbone; see Fig. 3 ▶ *b*, Supplementary Fig. S2). The strict conservation of this residue suggests that it plays an important role in aiding base recognition. The second stripe consists of the positively charged residues at position 8 (Lys/Arg), which are aligned along the noncoding strand phosphates (3.3–4.0 Å distance; Fig. 3 ▶ *b*, Supplementary Fig. S2). In contrast to the TALEs, which only interact with their coding strand, the presence of the second electropositive stripe on the surface of BurrH (Supplementary Fig. S2) determines the interaction of the repeat array with both strands of its DNA target (Supplementary Fig. S6).

### 3.3. BurrH DNA recognition   

The overall helix–loop–helix topology of the BuD repeats is reminiscent of those of TALEs (Deng *et al.*, 2012 ▶; Mak *et al.*, 2012 ▶; Stella *et al.*, 2013 ▶), yet the DNA-binding properties of BuD are different, consistent with its different amino-acid sequence (Figs. 1 ▶ *b*–1 ▶ *e*, Supplementary Figs. S2 and S6*a*). In contrast to TALE repeats, where the only sequence differences reside nearly exclusively in the RVDs (repeat variable dipeptides), the BuD repeats display higher sequence variability (Supplementary Fig. S2). The TALE RVDs determine nucleotide recognition; however, the corresponding loops in the BuD repeats, which are also involved in DNA-specific contacts, show differences at only a single residue. The first amino acid in their loops (position 12 in the repeat) is a conserved Asn, which is engaged in an interaction with the main chain of the residue at position 8 in the same repeat (Supplementary Fig. S7*a*). Besides Asn, TALE repeats can display a His in this position with a similar intra-repeat association (Deng *et al.*, 2012 ▶; Mak *et al.*, 2012 ▶; Stella *et al.*, 2013 ▶). Therefore, the BurrH–DNA complex suggests that this platform could be engineered following a single amino acid-to-nucleotide recognition code, and that BuD specificity is controlled by a single amino acid in this loop (position 13 in the repeats). Hence, this residue may constitute a BuD base-specifying residue (BSR) establishing a direct recognition code with the DNA (Supplementary Figs. S7*b*–S7*f*, Supplementary Table S2). To assess whether BuD can be specifically engineered using a simplified single-amino-acid code, we built arrays using the BSR code using only the residues at position 13 of the repeats (Supplementary Table S2). For this purpose, the His residues at position 12 of variants 1, 2, 3 and 4 were substituted by Asn (Supplementary Fig. S8). These refurbished BurrH variants were able to recognize and bind specifically to its DNA target, conserving their biophysical properties, demonstrating that this platform can be redesigned using the BSR code.

### 3.4. BuD repeats display new specific DNA interactions   

The BuD repeats present new interactions apart from the Ile–A, Asp–C, Asn–G, Gly–T and Ser–A interactions previously reported for TALE (Boch *et al.*, 2009 ▶; Deng *et al.*, 2012 ▶; Mak *et al.*, 2012 ▶). The 4th, 12th and 13th repeats in BurrH show new associations (Thr–A and Arg–G) involving bases in the coding and noncoding strands, respectively. These novel interactions expand the possibilities for targeting new sequences. Thr193 and Thr457 in BurrH associate with A_+4_ and A_+12_ in the coding strand (Fig. 3 ▶c). In the Thr–A association the side-chain methyl group makes van der Waals interactions with the purine rings. In the case of Thr193 the side chain also interacts with the preceding G_+3_ in the coding strand. Interestingly, the side-chain hydroxyl group makes a hydrogen bond to the side chain of Asn226 in the following BSR, generating a conformation that favours specific recognition of G_+5_ in the coding strand.

A striking interaction is observed for Arg490 in the 13th repeat with G_+5_ in the noncoding strand (Fig. 3 ▶ *d*). The guanidinium group of Arg490 builds a network of interactions with A_+14_ in the coding strand and T_+6_ and G_+5_ in the noncoding strand. This crossed interaction has never been observed in TALE, which exclusively associates with the coding DNA strand (Deng *et al.*, 2012 ▶; Mak *et al.*, 2012 ▶; Stella *et al.*, 2013 ▶) targeted by the protein. Thereby, BurrH recognizes bases in both DNA strands. The presence of one or more of these BSRs in tailored variants could aid in modulating the residence time in the binding site.

### 3.5. BurrH N-terminal region   

The N-terminal region of BurrH is in the neighbourhood of the DNA, thus we evaluated whether its two degenerate BuD repeats may influence the nucleotide preference in this area, as has been shown for TALE (Boch *et al.*, 2009 ▶). ITC measurements showed that this protein region does not show any DNA specificity (Supplementary Fig. S9). Finally, the C-terminal region contains another two degenerate repeats, which display a different primary structure yet conserve the topology (Supplementary Fig. S1). The first degenerate repeat contains Gly721 in the putative BSR; however, this residue does not contact T_+20_ (Supplementary Fig. S10*a*). In the final repeat the side chain of Arg753 disrupts the A–T pair, generating a hydrogen bond to T_−1_ in the noncoding strand and a cation–π interaction between its guanidinium group and the ring of T_+20_ in the coding strand (Supplementary Fig. S10*b*). Therefore, all BurrH arginines present at the 13th position of the BuD repeat interact with the noncoding-strand bases, suggesting that these amino acids may play an important role in DNA target recognition and could be employed to restrict the interaction of the protein with double-strand nucleic acids.

### 3.6. BurrH targeting *in vivo*   

**Figure 4:**
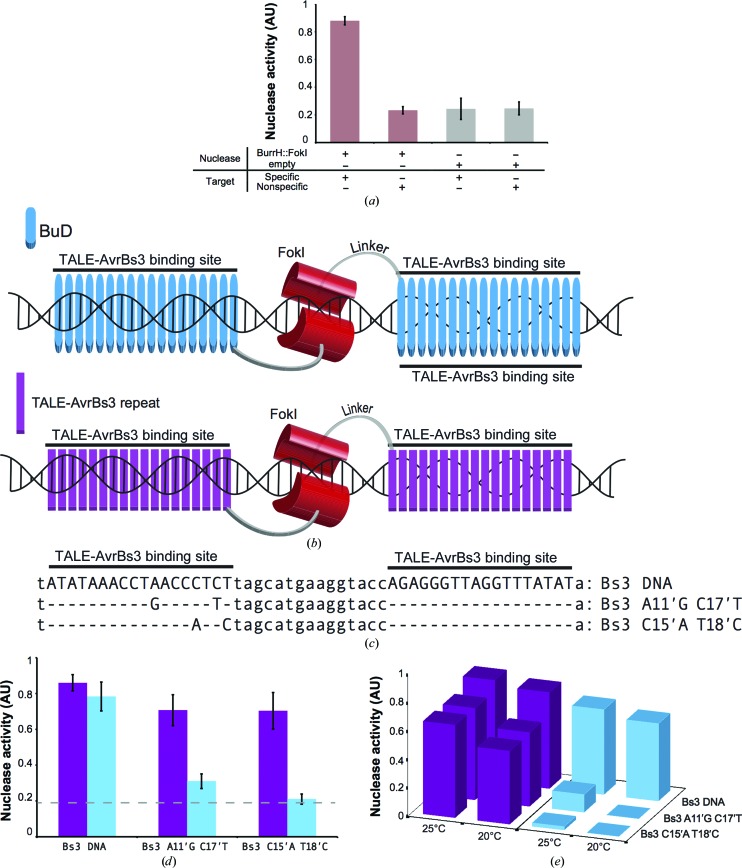
Engineered BuDNs can target a DNA sequence in a cellular scenario. (*a*) Nuclease activity of BuDN towards its homodimeric target in yeast. Upon mating, the BuDNs generate a double-strand break at the site of interest, allowing the restoration of a functional *lacZ* gene by single-strand annealing (SSA), enabling the generation of a blue colour in the presence of X-Gal. The colour was quantified and scored as an Afilter value, a parameter correlated to the nuclease activity. (*b*) Sketch of the BuDN design (see Supporting Information). A BuD array (cyan) targeting the desired DNA sequence was fused to FokI similarly to an AvrBs3-based TALEN (purple). (*c*) A pair of BuDNs targeting the AvrBs3 sequence (Bs3) was built to compare its activity with AvrBs3-based TALEN. The different DNA targets used in the assay are shown. The Bs3 DNA contains two identical Bs3 binding sites in opposite orientations separated by a 15 bp DNA spacer. Bs3 A11′G C17′T and C15′A T18′C DNAs contain two base-pair substitutions each in only one of the Bs3 binding sites. (*d*) Nuclease activity of the BuDNs and TALEN towards the DNA targets. The grey dashed line indicates the experimental background level. (*e*) Comparison of the nuclease activity of both scaffolds towards the same target at different temperatures. BuDNs are sensitive to variations in the target sequence, while TALEN seem to ignore the mutations in the DNA. The background level has been subtracted from the histograms. The obtained values are an average of three independent experiments. See Supporting Information for a detailed description of the nucleases.

All of the physicochemical properties of BurrH have been tested in a cellular scenario. We evaluated the performance of BurrH targeting its own DNA sequence by fusing the FokI nuclease domain to its C-terminal region, creating an artificial nuclease (BuDN; see *Methods* [Sec sec2] and Supporting Information for details). The activity was tested in a single-strand assay (SSA; Arnould *et al.*, 2006 ▶) in yeast, which relies on the restoration of a reporter gene after inducing a specific double-strand break (DSB) on the target DNA (Fig. 4 ▶ *a*). The generation of DSBs by the BurrH-derived nuclease on its target was very efficient (Fig. 4 ▶ *a*), demonstrating that this template can be employed to create precise DSBs in a cellular context.

### 3.7. Engineered BurrH targets the TALE sequence with high specificity   

We also assessed the engineering of BurrH in yeast to target a new DNA by creating a directed artificial nuclease (Fig. 4 ▶ *b*). To compare the properties of this new DNA-targeting platform with its cousin scaffold, the standard TALEN tools, we engineered the repeat array of BurrH using the four commonly used RVDs from AvrBs3-TALE (Boch *et al.*, 2009 ▶; Moscou & Bogdanove, 2009) and removing the 13th and 14th modules to target the 2 bp shorter sequence of AvrBs3 (Fig. 4 ▶ *c*, see *Methods* [Sec sec2] and Supporting Information for details). The direct comparison of HD and ND RVDs in the context of TALE has already been reported (Cong *et al.*, 2012 ▶). Thus, we only replaced the ND di-residue found in the native BurrH protein by the HD from AvrBs3 to target the cytosine nucleotide, ‘TALEnizing’ BurrH for direct comparison. The nuclease activities of the TALEN and BuDN nucleases were quantified using the SSA assay at 298 K. In addition to the AvrBs3 pseudo-palindromic target (the two duplicated AvrBs3 target sequences in inverse orientation are facing each other, separated by the so-called sequence spacer), we examined two additional targets containing two mutations on one side (Fig. 4 ▶ *c*). We then assessed the nuclease activity of BuDN and TALEN on these targets. Our results show that the activity of the BuDN was high and similar to the TALEN on its wild-type target, demonstrating that the engineered BuD proteins are able to recognize a new DNA sequence delivering effector proteins accurately on the DNA target (Fig. 4 ▶ *d*). Remarkably, BuDN activity decreased in the mutated targets (Fig. 4 ▶ *d*). Changes in only two bases can severely affect BuDN activity, while TALEN seems not to be sensitive to those variations. Furthermore, to investigate whether the particular thermodynamic properties of the BuD array can be exploited to improve its specificity, we performed the SSA assay at 298 and 293 K (Fig. 4 ▶ *e*). A comparison between the two assays shows that while TALENs were almost insensitive both in activity and specificity to the temperature decrease, BuDNs displayed a slight reduction in activity. However, the specificity of the BuDNs was high and the activity was reduced at 298 K and almost abolished at 293 K in the assay targeting the DNAs with only two mutations. Given the fact that BuD arrays achieve DNA binding through an entropic optimization, their temperature dependence is stronger than that observed in TALE, where DNA binding is enthalpy-driven (Stella *et al.*, 2013 ▶), thus improving its targeting specificity. Hence, this scaffold could offer the possibility of performing certain applications at lower temperatures to increase the accuracy in target recognition with a minor cost in activity. This property might represent a very important asset for *ex vivo* applications, which could be achieved at low temperatures, increasing targeting specificity.

### 3.8. BuD arrays target human genes with high efficiency   

**Figure 5:**
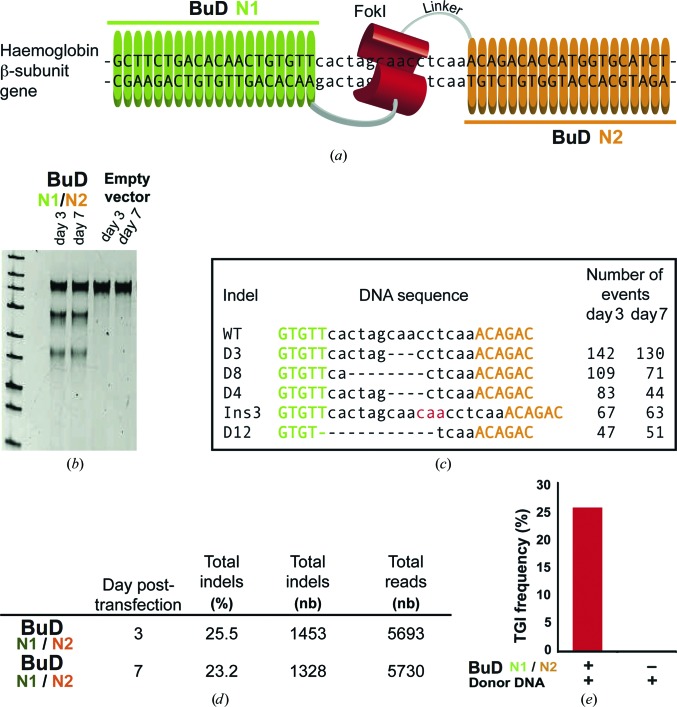
BuDNs targeting the HBB gene are accurate and highly active. (*a*) Two BuD arrays targeting a DNA region within the HBB gene near a locus known to be responsible for sickle-cell anaemia were generated. The arrays were fused to a FokI domain and transfected into HEK293 human cells (see *Methods* [Sec sec2]). (*b*) The efficiency of the double-strand breaks induced by the BuDNs was monitored using the T7 endonuclease assay. (*c*) The genomic DNA was also analyzed by deep sequencing. The most representative indels identified are reported in the table. Insertions are depicted in red and deletions by dashes. (*d*) Table quantifying the targeted mutagenesis events at the HBB locus by deep sequencing (see Supporting Information). (*e*) Targeted gene insertion (TGI) frequency determined at the HBB locus in the presence of the donor DNA transfected with or without BuDNs. See Supplementary Fig. S11 and Supporting Information for a detailed description.

After analyzing the efficiency and versatility of BuDs through the activity of BuDNs in yeast, we evaluated their performance in a genome-editing application in human cells. BuDNs were tailored to specifically target a DNA region near an area known to contain mutations responsible for sickle-cell anaemia in the human haemoglobin β (HBB) gene (Fig. 5 ▶ *a*). We generated BuDN1 and BuDN2, which were transfected into HEK293 human cells. The efficiency of the BuDN1/N2 in inducing double-strand-break events was calculated using a T7 endonuclease assay (Reyon *et al.*, 2012 ▶; Valton *et al.*, 2012 ▶) to measure the level of indels (insertion and deletion events) generated by the nonhomologous end-joining (NHEJ) repair pathway (Fig. 5 ▶ *b*). These data clearly indicated that BuDN1/N2 was able to generate strong levels of targeted mutagenesis at the HBB locus 3 and 7 d post-transfection. The nature and the frequency of the indels were further investigated by amplicon deep sequencing (Figs. 5 ▶ *c* and 5 ▶ *d*). Remarkably, these nucleases induced efficient mutagenesis at up to 25.5% 3 d post-transfection, which was stable over time, and at up to 23.2% 7 d post-transfection (ratio D7/D3 = 0.91). These frequencies obtained with the BuD platform are similar to those observed for fully engineered TALENs in 84 different human genes using a similar approach (Reyon *et al.*, 2012 ▶). Moreover, the persistence of these events over time suggests that BuD arrays are a safe scaffold for genome modification.

Finally, to evaluate the potential of BurrH for targeted gene insertion (TGI) experiments in a homologous recombination (HR)-based strategy, we monitored the specific insertions of a sequence of 29 base pairs at the same HBB locus induced by our BuDN1/N2 18 d post-transfection. To perform this experiment, we designed a plasmid-based donor DNA that contained two homology arms of 1193 and 959 bp surrounding the inserted sequence. With this design, we were able to achieve high levels of TGI (up to 25%; Fig. 5 ▶ *e*) induced by this nuclease 18 d post-transfection, suggesting that this template displays low cytotoxicity, presumably owing to its good DNA specificity, avoiding the risk of inducing deleterious levels of mutagenesis as has been shown for RNA-guided nucleases (Fu *et al.*, 2013 ▶). Overall, the BurrH-based nuclease presented *in vivo* activity levels that are compatible with genome-editing applications. These promising results suggest that these variants may improve the success of different applications, including difficult applications such as gene repair, which may be employed in the treatment of monogenic diseases.

## 4. Discussion   

We have dissected the DNA-binding properties of BurrH, providing a rational basis for its redesign to target new sequences. Changes in only the 13th residue of the BuD repeat can be employed to target new sequences. A structural comparison of BurrH protein with TALEs suggests that protein evolution has generated a structural helix–loop–helix motif to create a modular DNA-binding domain. The ample versatility of this structural element to recognize nucleic acids has also been developed to produce modular single-strand RNA-binding units such as the PPR proteins (Yin *et al.*, 2013 ▶). Most likely, in the case of the DNA-binding proteins this common template was developed from the spatial restrictions imposed by the double helix. However, the different amino-acid combinations expand the possibilities for accommodating specific DNA binding while conserving the structure of the domain. BuD arrays are the first modular helix–loop–helix domains containing nonrepetitive sequences that have been used in genome editing. This represents an advantage with respect to TALE, whose DNA sequences are prone to rearrangements when delivered by lentivirus in target cells owing to their highly repetitive DNA sequence (Holkers *et al.*, 2013 ▶). The features of BurrH and its customization for genome-editing applications might make this protein an excellent platform for the engineering of novel DNA specificities, and besides other applications this template may be well suited for the development of specific modulators of transcription fused to the corresponding effector domains (Miller *et al.*, 2011 ▶). Our work shows that this novel platform can be engineered to recognize DNA sequences in human cells. Hence, the combination of the efficiency and specificity of BuD is well suited to push forward multiple genome-modification approaches for cell or organism redesign, opening new avenues for precise and safe gene editing for biomedical purposes.

## Supplementary Material

Supplementary Tables and Figures.. DOI: 10.1107/S1399004714011183/cb5061sup1.pdf

Supplementary Movie 1. Video of the conformational change between the apo and DNA-bound forms.. DOI: 10.1107/S1399004714011183/cb5061sup2.mov

Detailed description of the deep sequencing results (Fig. 5).. DOI: 10.1107/S1399004714011183/cb5061sup3.xlsx

PDB reference: apo BurrH, 4cj9

PDB reference: BurrH–DNA complex, 4cja

## Figures and Tables

**Table 1:** Data-collection, phasing and refinement statistics Values in parentheses are for the highest resolution shell. One crystal was used to solve each structure.

	Apo BurrH	BurrHDNA
Data collection		
Space group	*P*3_1_	*P*2_1_
Unit-cell parameters (, )	*a* = *b* = 73.28, *c* = 268.02, = = 90, = 120	*a* = 70.15, *b* = 95.83, *c* = 76.61, = = 90, = 109.51
Wavelength ()	0.98	1.00
Resolution ()	46.082.21 (2.332.21)	47.922.65 (2.792.65)
*R*_merge_ †	0.11 (0.42)	0.07 (0.61)
*R*_meas_	0.13 (0.49)	0.08 (0.72)
No. of reflections	79917	28134
Mean *I*/(*I*)	7.0 (2.4)	12.1 (1.7)
Completeness (%)	98.7 (99.8)	97.7 (99.9)
Multiplicity	3.5 (3.4)	3.4 (3.4)
SAD phasing		
No. of Se sites found	12/12	
FOM	0.45	
Phasing power	1.8	
Refinement		
Resolution ()	63.502.21	38.662.65
No. of reflections	79812	27719
*R*_work_/*R*_free_	0.18/0.23	0.20/0.27
No. of molecules in asymmetric unit	2	1
No. of atoms		
Protein	10970	5491
Ligand/ion	0	936
Water	953	129
R.m.s. deviations		
Bond lengths ()	0.003	0.008
Bond angles ()	0.712	1.366
Average *B* factor (^2^)	39.02	65.54
Ramachandran plot		
Favoured (%)	99.73	93.48
Allowed (%)	0.27	6.13
Outliers (%)	0.00	0.40

† *R*_merge_ is defined according to Kabsch (2010 ▶).

## Footnotes

1 Supporting information has been deposited in the IUCr electronic archive (Reference: CB5061).
