# Supplementary material for: BuD, a helix–loop–helix DNA-binding domain for genome modification
Source: Acta Crystallogr D Biol Crystallogr. 2014 Jun 29;70(Pt 7):2042–52. doi: 10.1107/S1399004714011183 (PMC4089491; doi:10.1107/S1399004714011183)
Supplement: Supplementary file 1 [file d-70-02042-sup1.pdf]

SUPPLEMENTARY RESULTS, TABLES & FIGURES

BuD, a helix-loop-helix DNA-binding domain for genome modification

Stefano Stella[1,2#], Rafael Molina[1#], Blanca López-Méndez[3], Alexandre Juillerat[4], Claudia Bertonati[4], Fayza Daboussi[4], Ramon Campos-Olivas[3], Phillippe Duchateau[4] and Guillermo Montoya[1,2]*

[1]Structural Biology and Biocomputing Programme, Spanish National Cancer Research Centre (CNIO), Macromolecular Crystallography Group, [2]Structural Biology Group, Novo Nordisk Foundation Center for Protein Research, Faculty of Health and Medical Sciences, University of Copenhagen, Blegdamsvej 3B, 2200 Copenhagen, Denmark, [3]Spectroscopy and NMR Unit, c/Melchor Fdez. Almagro 3, 28029 Madrid, Spain. [4]Cellectis, 8 Rue de la Croix Jarry, 75013 Paris, France.

[#]Contributed equally.
*To whom correspondence should be addressed Email:
guillermo.montoya@cpr.ku.dk

Index

Supplementary Results

Detailed description of the engineered nucleases

Supplementary Tables

Supplementary Figures

SUPPLEMENTARY RESULTS

Detailed description of the BurrH apo and protein-DNA bound structures

BurrH apo structure is arranged in a continuous right-handed superhelical assembly (Fig. 2a). The structurally well-defined region of DNA-free BurrH (residues 15 to 766) is composed by 19 repeats located at the central region (residues 82 to 708) plus two-degenerated BuD repeats at the N- (residues 15 to 81) and C-terminal (residues 709 to 766) regions (Supp. Fig. 1). The overall dimensions of the structure are approximately 60 Å by 60 Å by 110 Å (Fig. 2a). BurrH N-terminal region is much shorter and well structured compared to the TALEs. Each BuD repeat in BurrH contains 33 amino acids, with residues 3 to 10 forming a short alpha helix and residues 15 to 32 constituting an extended kinked alpha helix. The short helices form an internal surface along the superhelical axis, whereas the kinked helices constitute an external surface (Fig. 2a). The two helices are connected by a short loop consisting of two glycines at position 11 and 14 in the repeat, one Asn at position 12, and the residue at position 13 that constitutes the BSR (BuDs, base-specifying residue, see Supp. Fig. 2). A detailed secondary structure analysis (DSSP) shows that some of the BuD repeats presented 3_{10} helices at the end of the kinked helix (repeats 1, 13 and 17). Despite the sequence variability in the repeats, the conservation of some residues in key positions results in their similar conformation. Short and kinked helices within each repeat closely pile against each other through extensive van der Waals contacts. Apo BurrH shows a left-handed packing of the consecutive helices within and between the individual repeats conforming the superhelical structure previously described. In contrast with TALE proteins, BurrH C-terminal region is shorter and lacks both a nuclear localization signal and a transcription activation domain. Instead, it is composed of two repeats; the first is a slightly degenerated BuD repeat (residues 709 to 740) while the second shows a higher degree of variability (residues 741 to 766).

The BurrH-DNA complex structure follows the previously described folding of the apo protein, wrapping around the DNA double helix. The DNA structure displays an almost unperturbed B-form DNA, with the exception of the last bp close to the C-terminal region where Arg753 disrupt the duplex DNA contacting with a T_{+21} and displacing its complementary adenine base. We were able to build unambiguously the model from protein residues 15 to 767 and the 23 bp of the DNA target. The superhelical BurrH structure follows the major groove of the DNA duplex. Similar to DNA-free BurrH, all repeats exhibit a nearly identical conformation except the loops

containing the BSR (BuD base-specifying residue) in the 3rd and 13th repeats, which in the BurrH-DNA complex correspond to an atypical long Asn-$G_{+3(coding)}$ interaction (4.1 Å) and to the novel and strong Arg-$G_{+15(non\ coding)}$ interaction, respectively (Supp. Fig 7c, Fig. 2f). Interestingly, in both cases their BSR containing loops suffer a rearrangement after DNA binding that disturbs not only the loop but also the secondary structure of the adjacent helices. Upon DNA binding, the superhelical pitch is reduced from 110 Å in DNA-free form to 87 Å in the DNA-bound form (Fig. 2a). While the first two thirds of the main chain repeats superimpose well, small conformational variations are accumulated resulting in notable differences between the positions of the Cα atoms in Gly33. Such differences are gradually amplified over an increasing number of repeats resulting in the compression of the superhelical assembly in the DNA-bound form. Such conformational plasticity is consistent with the van der Waals interactions between adjacent BuD repeats, which can tolerate minor distance shifts. Remarkably, in BurrH the superhelical compression is aided in some repeats by a strong electrostatic interaction carried out between the arginines in position 32 and the glutamic acids at position 26 in the repeats (Supp. Fig. 2). The arrangement is different to the inter-repeat interactions in observe in TALEs such as AvrBs3. The intra-repeat interactions in BurrH are driven mainly by van der Waals contacts, but while in TALEs such as AvrBs3 there is a hydrophobic interaction between the positions 1 and 26, in BurrH this interaction is absent. Interestingly, in BurrH most of the hydrophobic core interactions are maintained (positions 6-9-19 and 1-22) except the interaction 1-26 due to lack of a hydrophobic residue at position 26 in the BuD repeats. Interestingly, after superimposing the repeats of BurrH, which are shorter in length (33-aa), with the classical 34-aa repeats of TALE, differences are observed between the positions of the Cα atoms in the last residues of the repeat.

Electrostatic potential and DNA binding in BurrH

In contrast with TALEs, which only recognize one of the strands (herein referred to as the coding strand), the BuD helix-loop-helix motif establishes a network of protein-DNA contacts with both strands complementing the direct recognition code. The electrostatic potential of BurrH shows two electropositive stripes running along the protein and contacting the phosphate backbones of the double helix (Supp. Fig. 6). The coding strand interacts with one of these stripes composed of a conserved Gln in position 17, whose conformation is favored by the presence of two-conserved Gly residues and one Ala residue located after the BSR in positions 14th, 15th and 16th in the repeat (Supp. Fig. 1). Their strict conservation in BuD repeats and TALEs

suggests that these residues may play an important role helping base recognition by the BSR. The second stripe consists of the positive charged residues in position 8th (Lys/Arg), which are aligned along the non-coding strand phosphates (3.5-4.0 Å distances). Noteworthy, the 8th position is occupied by an Ala in the TALE and the Lys is located in position 16th of the repeats, right before the conserved Gln. The exchange of these residues in the BuD repeats (Supp. Fig. 2) creates a new electropositive band contacting the non-coding strand and determining the interactions of these repeat arrays with both strands of its DNA target.

Detailed description of the BSR-base interaction

The invariant Asn at position 12th in BuD repeats interacts with the main chain carbonyl group of the residue at position 8th in the same motif (Supp. Fig 7a), resulting in a C-capping of the first α-helix of the BuD repeat. The following residue at position 13th is involved in base recognition and constitutes the BSR. BurrH displays six Ile-BSRs (associated to adenosines), four Asn-BSRs (associated to guanosines), two Thr-BSRs (associated to adenosines), two Asp-BSRs (associated to cytosines), two Ser-BSRs (associated to adenosines), two Gly-BSRs (associated to thymines) and one Arg-BSR (associated to guanosine in the non-coding strand) (Fig 3, Supp. Fig. 7b-e). Each of these different BSRs to nucleotide base interaction is described below.

Ile-BSRs

The aliphatic side chain of the isoleucine residue makes van der Waals contacts to C8 (and N7) of the adenine purine ring (Supp. Fig. 7b).

Gly-BSRs

The lack of side-chain allows the Cα of the glycine residue to associate through van der Waals forces with the methyl group of the thymine base (Supp. Fig. 7e).

Asn-BSRs

The asparagine is positioned to form hydrogen bond with the N7 of the guanine base. Interestingly, in repeats 3 and 5 Asn-BSRs show an interaction distance of 4.1 Å, while repeats 8 and 16 asn-BSRs display an interaction distance of 3.0 Å (Supp. Fig. 7c).

Thr-BSRs

In the Thr-A association the methyl group of the Thr193 and Thr457 located in the fourth BSR makes van der Waals interactions with the purine rings of A_{+4} and $A_{+12.}$ In the Thr-A association the methyl group of the Thr193 and Thr457 located in the 4th and 12th BSR makes van der Waals interactions with the purine rings of A_{+4} and $A_{+12.}$ Interestingly, the side chain hydroxyl group of Thr193 makes a hydrogen bond with the side chain of Asn226 in the following BSR, generating a conformation that favors its specific recognition of G_{+5} in the coding strand (Fig. 3c).

Asp-BSRs

The Asp-C association in the 9th and 15th BuD repeat is defined by the interaction of the C amine group by the side chain of the Asp residue. Again the conformation of Asn325 in the previous BSR (in the case of the 9th repeat) could influence the side chain conformation (Supp. Fig. 7d).

Ser-BSRs

The serine hydroxyl group donates a hydrogen bond to the N7 atom of adenine (Supp. Fig. 5e). In the R-BSR repeat, the Arg interacts with the adenine at position 14 and with the thymine and guanine at positions 6 and 5 of the non-coding strand, respectively (Supp. Fig. 7f).

Recognition of the nucleotide in position 0

The N-terminal region of TALEs has been proposed to impose the presence of a thymine (named T_0) in the target DNA position that precedes the first nucleotide recognized by the canonical repeat sequence. BurrH does not show preference for any nucleotide in this position (Supp. Fig. 9). The N-terminal section reveals two degenerated repeat folds. We termed these -1 and 0 repeats (Supp. Fig. 1) composed of residues 17–48 and 49–81, respectively. No detectable sequence identity can be found between these two cryptic modules and the N-terminal TALE repeats reported. The interaction between T_0 and the N-terminal region of BurrH occurs through van der Waals interactions between the methyl groups of the T_0 base and C_β of the D61 residue. The same type of association is observed for T_{-1} and the aromatic ring of Tyr29.

Detailed description of the engineered nucleases

Amino acid sequences of the engineered BurrH based nucleases used for the celullar experiments in Fig. 4 and Fig. 5.

(Fig. 4a)

Amino acid sequences of BurrH::FokI for SSA assay targeting BurrH binding site

MGDPKKKRKVIDYPYDVPDYA NLS and HA Tag

IDIASTAFVDQDKQMANRLNLSPLERSKIEKQYGGATTLAFI **BurrH**
SNKQNELAQILSRADILKIASYDCAAHALQAVLDCGPMLGKRG **N-terminal**

	BuDs	Base recognized
FSQSDIVKIAGNIGGAQALQAVLDLESMLGKRG	1	A
FSRDDIAKMAGNIGGAQTLQAVLDLESAFRERG	2	A
FSQADIVKIAGNNGGAQALYSVLDVEPTLGKRG	3	G
FSRADIVKIAGNTGGAQALHTVLDLEPALGKRG	4	A
FSRIDIVKIAANNGGAQALHAVLDLGPTLRECG	5	G
FSQATIAKIAGNIGGAQALQMVLDLGPALGKRG	6	A
FSQATIAKIAGNIGGAQALQTVLDLEPALCERG	7	A
FSQATIAKMAGNNGGAQALQTVLDLEPALRKRD	8	G
FRQADIIKIAGNDGGAQALQAVIEHGPTLRQHG	9	C
FNLADIVKMAGNIGGAQALQAVLDLKPVLDEHG	10	A
FSQPDIVKMAGNIGGAQALQAVLSLGPALRERG	11	A
FSQPDIVKIAGNTGGAQALQAVLDLELTLVEHG	12	A
FSQPDIVRITGNRGGAQALQAVLALELTLRERG	13	T
FSQPDIVKIAGNSGGAQALQAVLDLELTFRERG	14	A
FSQADIVKIAGNDGGTQALHAVLDLERMLGERG	15	C
FSRADIVNVAGNNGGAQALKAVLEHEATLNERG	16	G
FSRADIVKIAGNGGGAQALKAVLEHEATLDERG	17	T
FSRADIVRIAGNGGGAQALKAVLEHGPTLNERG	18	T
FNLTDIVEMAANSGGAQALKAVLEHGPTLRQRG	19	A
LSLIDIVEIASNGGAQALKAVLKYGPVLMQAG	20	T

RSNEEIVHVAARRGGAGRIRKMVAPLLERQ **BurrH C-terminal**

GRSGSDPISRSQLVKSELEEKKSELRHKLKYVPHEYIELIEIARN
STQDRILEMKVMEFFMKVYGYRGKHLGGSRKPDGAIYTVGSPIDY FokI domain
GVIVDTKAYSGGYNLPIGQADEMQRYVEENQTRNKHINPNEWWKV
YPSSVTEFKFLFVSGHFKGNYKAQLTRLNHITNCNGAVLSVEELL
IGGEMIKAGTLTLEEVRRKFNNGEINFAAD

DNA target in this SSA assay

AAGAGAAGCAAATACGTTAT tagcatgaaggtacc ATAACGTATTTGCTTCTCTT

(Fig. 4 d-e)

Amino acid sequences of BuD-AvrBs3::FokI for SSA assay

MGDPKKKRKVIDYPYDVPDYA NLS and HA Tag

IDIASTAFVDQDKQMANRLNLSPLERSKIEKQYGGATTLAFI **BurrH**
SNKQNELAQILSRADILKIASYDCAAHALQAVLDCGPMLGKRG **N-terminal**

Sequence	BuDs	Base recognized
FSQSDIVKIAGHDGGAQALQAVLDLESMLGKRG	1	A
FSRDDIAKMAGNGGGAQTLQAVLDLESAFRERG	2	T
FSQADIVKIAGNIGGAQALYSVLDVEPTLGKRG	3	A
FSRADIVKIAGNGGGAQALHTVLDLEPALGKRG	4	T
FSRIDIVKIAANIGGAQALHAVLDLGPTLRECG	5	A
FSQATIAKIAGNIGGAQALQMVLDLGPALGKRG	6	A
FSQATIAKIAGNIGGAQALQTVLDLEPALCERG	7	A
FSQATIAKMAGHDGGAQALQTVLDLEPALRKRD	8	C
FRQADIIKIAGHDGGAQALQAVIEHGPTLRQHG	9	C
FNLADIVKMAGNGGGAQALQAVLDLKPVLDEHG	10	T
FSQPDIVKMAGNIGGAQALQAVLSLGPALRERG	11	A
FSQPDIVKIAGNIGGAQALQAVLDLELTLVEHG	12	A
FSQADIVKIAGHDGGTQALHAVLDLERMLGERG	13	C
FSRADIVNVAGHDGGAQALKAVLEHEATLNERG	14	C
FSRADIVKIAGHDGGAQALKAVLEHEATLDERG	15	C
FSRADIVNVAGNGGGAQALKAVLEHEATLNERG	16	T
FNLTDIVEMAAHDGGAQALKAVLEHGPTLRQRG	17	C
LSLIDIVEIAGNGGGAQALKAVLKYGPVLMQAG	18	T

RSNEEIVHVAARRGGAGRIRKMVAPLLERQ **BurrH C-terminal**

GRSGSDPISRSQLVKSELEEKKSELRHKLKYVPHEYIELIEIARN
STQDRILEMKVMEFFMKVYGYRGKHLGGSRKPDGAIYTVGSPIDY FokI domain
GVIVDTKAYSGGYNLPIGQADEMQRYVEENQTRNKHINPNEWWKV
YPSSVTEFKFLFVSGHFKGNYKAQLTRLNHITNCNGAVLSVEELL
IGGEMIKAGTLTLEEVRRKFNNGEINFAAD

DNA target in this SSA assay

ATATAAACCTAACCCTCT tagcatgaaggtacc AGAGGGTTAGGTTTATAT

(Fig. 5)
Amino acid sequences of BuDN1 and BuDN2 targeting the HBB locus

BuD1N

MGDPKKKRKVIDYPYDVPDYA NLS and HA Tag

IDIASTAFVDQDKQMANRLNLSPLERSKIEKQYGGATTLAFI
SNKQNELAQILSRADILKIASYDCAAHALQAVLDCGPMLGKRG **BurrH N-terminal**

	BuDs	Base recognized
FSQSDIVKIAGNNGGAQALQAVLDLESMLGKRG	1	G
FSRDDIAKMAGHDGGAQTLQAVLDLESAFRERG	2	C
FSQADIVKIAGNGGGAQALYSVLDVEPTLGKRG	3	T
FSRADIVKIAGNGGGAQALHTVLDLEPALGKRG	4	T
FSRIDIVKIAAHDGGAQALHAVLDLGPTLRECG	5	C
FSQATIAKIAGNGGGAQALQMVLDLGPALGKRG	6	T
FSQATIAKIAGNNGGAQALQTVLDLEPALCERG	7	G
FSQATIAKMAGNIGGAQALQTVLDLEPALRKRD	8	A
FRQADIIKIAGHDGGAQALQAVIEHGPTLRQHG	9	C
FNLADIVKMAGNIGGAQALQAVLDLKPVLDEHG	10	A
FSQPDIVKMAGHDGGAQALQAVLSLGPALRERG	11	C
FSQPDIVKIAGNIGGAQALQAVLDLELTLVEHG	12	A
FSQADIVKIAGHIGGTQALHAVLDLERMLGERG	13	A
FSRADIVNVAGHDGGAQALKAVLEHEATLNERG	14	C
FSRADIVKIAGNGGGAQALKAVLEHEATLDERG	15	T
FSRADIVNVAGNNGGAQALKAVLEHEATLNERG	16	G
FNLTDIVEMAANGGGAQALKAVLEHGPTLRQRG	17	T
FSRADIVNVAGNNGGAQALKAVLEHEATLNERG	18	G
FNLTDIVEMAANNGGAQALKAVLEHGPTLRQRG	19	T
LSLIDIVEIAGNNGGAQALKAVLKYGPVLMQAG	20	T

RSNEEIVHVAARRGGAGRIRKMVAPLLERQ **BurrH C-terminal**

GRSGSDPISRSQLVKSELEEKKSELRHKLKYVPHEYIELIEIARN
STQDRILEMKVMEFFMKVYGYRGKHLGGSRKPDGAIYTVGSPIDY FokI domain
GVIVDTKAYSGGYNLPIGQADEMQRYVEENQTRNKHINPNEWWKV
YPSSVTEFKFLFVSGHFKGNYKAQLTRLNHITNCNGAVLSVEELL
IGGEMIKAGTLTLEEVRRKFNNGEINFAAD

BuD2N

MGDPKKKRKVIDYPYDVPDYA NLS and HA Tag

IDIASTAFVDQDKQMANRLNLSPLERSKIEKQYGGATTLAFI
SNKQNELAQILSRADILKIASYDCAAHALQAVLDCGPMLGKRG **BurrH N-terminal**

	BuDs	Base recognized
FSQSDIVKIAGNIGGAQALQAVLDLESMLGKRG	1	A
FSRDDIAKMAGNNGGAQTLQAVLDLESAFRERG	2	G
FSQADIVKIAGNIGGAQALYSVLDVEPTLGKRG	3	A
FSRADIVKIAGNGGGAQALHTVLDLEPALGKRG	4	T
FSRIDIVKIAANNGGAQALHAVLDLGPTLRECG	5	G
FSQATIAKIAGHDGGAQALQMVLDLGPALGKRG	6	C
FSQATIAKIAGNIGGAQALQTVLDLEPALCERG	7	A
FSQATIAKMAGHDGGAQALQTVLDLEPALRKRD	8	C
FRQADIIKIAGHDGGAQALQAVIEHGPTLRQHG	9	C
FNLADIVKMAGNIGGAQALQAVLDLKPVLDEHG	10	A
FSQPDIVKMAGNGGGAQALQAVLSLGPALRERG	11	T
FSQPDIVKIAGNNGGAQALQAVLDLELTLVEHG	12	G
FSQADIVKIAGNNGGTQALHAVLDLERMLGERG	13	G
FSRADIVNVAGNGGGAQALKAVLEHEATLNERG	14	T
FSRADIVKIAGNNGGAQALKAVLEHEATLDERG	15	G
FSRADIVNVAGNGGGAQALKAVLEHEATLNERG	16	T
FNLTDIVEMAAHDGGAQALKAVLEHGPTLRQRG	17	C
FSRADIVNVAGNGGGAQALKAVLEHEATLNERG	18	T
FNLTDIVEMAANNGGAQALKAVLEHGPTLRQRG	19	G
LSLIDIVEIAGNGGGAQALKAVLKYGPVLMQAG	20	T

RSNEEIVHVAARRGGAGRIRKMVAPLLERQ **BurrH C-terminal**

GRSGSDPISRSQLVKSELEEKKSELRHKLKYVPHEYIELIEIARN
STQDRILEMKVMEFFMKVYGYRGKHLGGSRKPDGAIYTVGSPIDY FokI domain
GVIVDTKAYSGGYNLPIGQADEMQRYVEENQTRNKHINPNEWWKV
YPSSVTEFKFLFVSGHFKGNYKAQLTRLNHITNCNGAVLSVEELL
IGGEMIKAGTLTLEEVRRKFNNGEINFAAD

DNA target
GCTTCTGACACAACTGTGTT cactagcaacctcaa ACAGACACCATGGTGCATCT

SUPPLEMENTARY TABLES

Supp. Table I.
Binding sequence of the oligonucleotides used to amplify the HBB locus in targeted mutagenesis experiments and to identify Knock-in positive clones in Targeted Gene Insertion experiments.

Targeted mutagenesis	Forward	5’-ccacaccctagggttggccaatctactccc-3’
	Reverse	5’-GTAGACCACCAGCAGCCTAAGGGTGGG-3’
Tageted Gene Insertion	Forward	5’-GGGATGGGAGAAAGGCGATCACGTTG-3’
	Reverse	5’-AATTGCGGCCGCGGTCCGGCGC-3’

Supp. Table II. *BuD repeat single amino acid to nucleotide code.*

BSR	Nucleotide
Ile	A
Thr	
Ser	
Asp	C
Arg	G non-coding strand
Asn	G
Gly	T

```
N-t                                        ANRLN         16

-1    LSPLERSKIEKQYGGATTLAFISN-KQNELAQI  T     48
 0    LSRADILKIASYDCAAHALQAVLDCGPMLGKRG  T0    81

 1    FSQSDIVKIAGNIGGAQALQAVLDLESMLGKRG  A     114
 2    FSRDDIAKMAGNIGGAQTLQAVLDLESAFRERG  A     147
 3    FSQADIVKIAGNNGGAQALYSVLDVEPTLGKRG  G     180
 4    FSRADIVKIAGNTGGAQALHTVLDLEPALGKRG  A     213
 5    FSRIDIVKIAANNGGAQALHAVLDLGPTLRECG  G     246
 6    FSQATIAKIAGNIGGAQALQMVLDLGPALGKRG  A     279
 7    FSQATIAKIAGNIGGAQALQTVLDLEPALCERG  A     312
 8    FSQATIAKMAGNNGGAQALQTVLDLEPALRKRD  G     345
 9    FRQADIIKIAGNDGGAQALQAVIEHGPTLRQHG  C     378
10    FNLADIVKMAGNIGGAQALQAVLDLKPVLDEHG  A     411
11    FSQPDIVKMAGNIGGAQALQAVLSLGPALRERG  A     444
12    FSQPDIVKIAGNTGGAQALQAVLDLELTLVEHG  A     477
13    FSQPDIVRITGNRGGAQALQAVLALELTLRERG  T     510
14    FSQPDIVKIAGNSGGAQALQAVLDLELTFRERG  A     543
15    FSQADIVKIAGNDGGTQALHAVLDLERMLGERG  C     576
16    FSRADIVNVAGNNGGAQALKAVLEHEATLNERG  G     609
17    FSRADIVKIAGNGGGAQALKAVLEHEATLDERG  T     642
18    FSRADIVRIAGNGGGAQALKAVLEHGPTLNERG  T     675
19    FNLTDIVEMAANSGGAQALKAVLEHGPTLRQRG  A     708

20    LSLIDIVEIASNGG-AQALKAVLKYGPVLMQAG  T     740
      RSNEEIVHVAARRGGAGRIRKMVAPLLERQ     A     770
      GGSEFELENLYFQGELRRQASALE           C-t   794
```

X BSR
X BSR-like residue

Supp. Figure 1.- Sequence of the crystallized BurrH protein showing the degenerated repeats in the N and C-terminal regions and the 19 BuD repeats (BSR, Base Specifying Residue). The target DNA sequence is shown on the right side and the repeat number is depicted on the left side.

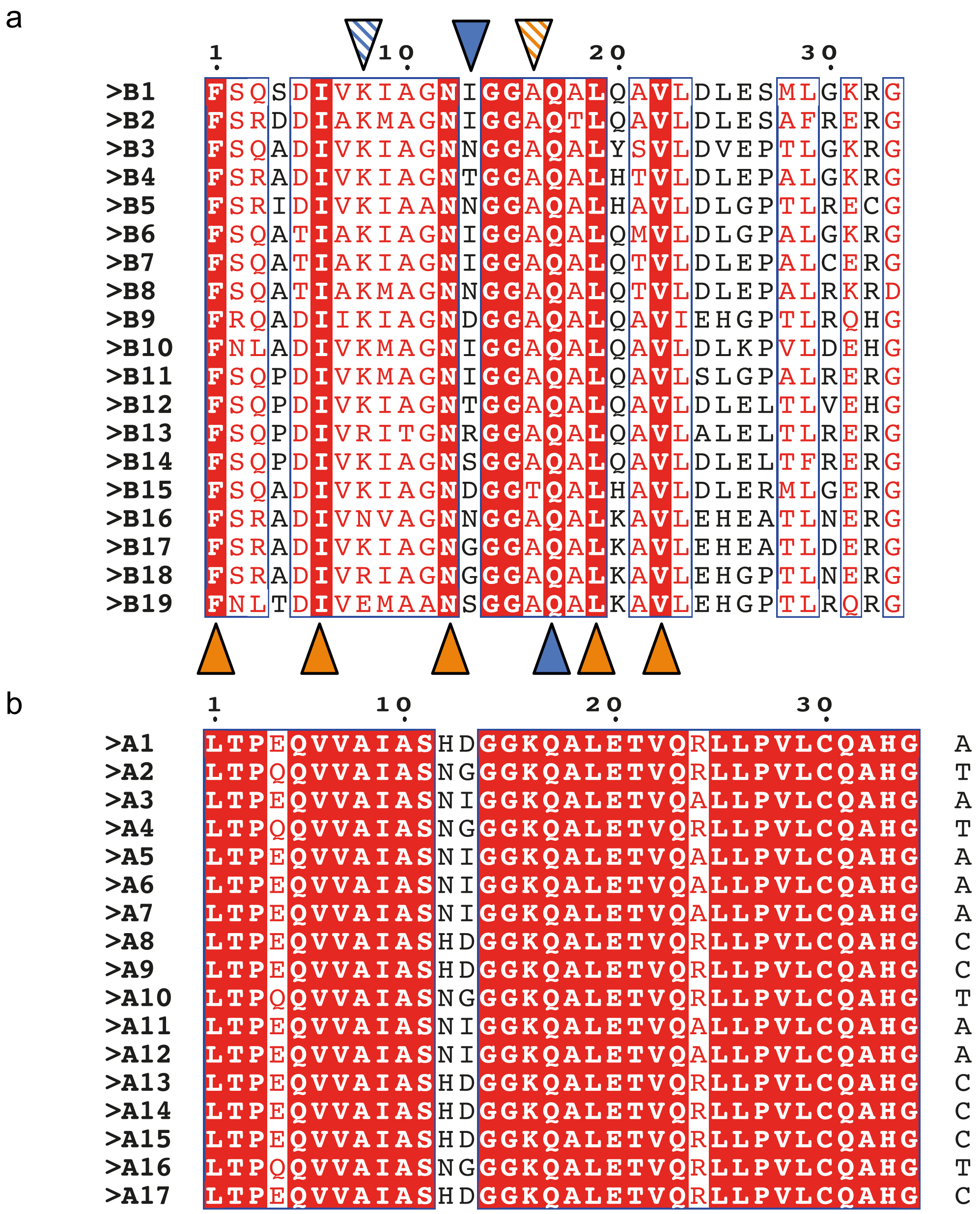

Supp. Figure 2.- Sequence alignments of BurrH and TALE repeats. **a)** BurrH and **b)** AvrBs3 TALE repeats alignment showing the identity of the conserved amino acids (red background). The AvrBs3 target is indicated on the right side. The residues involved in DNA recognition and phosphate backbone interaction are indicated (top and bottom blue triangle). The conserved residues involved in structural inter and intra repeat interactions are also indicated (orange triangle). The exchange of positions between the residues in the 8th and 16th positions respect to the TALE repeats is also labeled (blue dashed triangle for the polar and orange dashed triangle for the hydrophobic). The repeat number is on the left and the amino acid repeat position in each repeat on top.

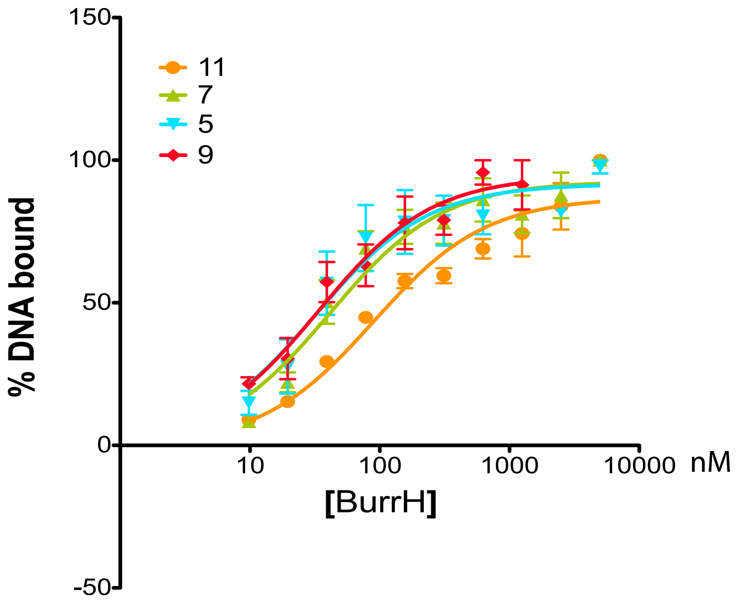


```
      I  I  N  T  N  I  I  N  D  I  I  T  R  S  D  N  G  G  S          BSR
5′-tt A  A  G     G  A  A  G  C  A  A        A  C  G  T  T  A   ta-3′  coding strand
```

Target	Sequences	K_D (nM)
1	- - - A - - - - - - - A A - - - - - - -	40 ± 3
2	- - - C - - - - - - - C A - - - - - - -	44 ± 9
3	- - - G - - - - - - - G A - - - - - - -	81 ± 12
4	- - - T - - - - - - - T A - - - - - - -	61 ± 8
5	- - - A - - - - - - - A T - - - - - - -	32 ± 7
6	- - - C - - - - - - - C T - - - - - - -	43 ± 10
7	- - - G - - - - - - - G T - - - - - - -	42 ± 7
8	- - - T - - - - - - - T T - - - - - - -	nd
9	- - - A - - - - - - - A G - - - - - - -	35 ± 6
10	- - - C - - - - - - - C G - - - - - - -	49 ± 10
11	- - - G - - - - - - - G G - - - - - - -	90 ± 12
12	- - - T - - - - - - - T G - - - - - - -	63 ± 5
13	- - - A - - - - - - - A C - - - - - - -	39 ± 10
14	- - - C - - - - - - - C C - - - - - - -	47 ± 5
15	- - - G - - - - - - - G C - - - - - - -	59 ± 6
16	- - - T - - - - - - - T C - - - - - - -	nd

Supp. Figure 3.- Base specificity of threonine and arginine in position 13th of BuD repeats. The base preference of the motif containing Thr and Arg in the 13th position (4th, 12th, and 13th repeats) was tested using different 23 base pair DNA duplexes (only the coding strand is shown), containing each of the four bases in each of the three corresponding positions in the DNA. Dissociation constants (K_D) for 14 out of the 16 possible combinations of the DNA target were measured by fluorescence anisotropy. The upper panel shows experimental data and non-linear fits to a 1:1 binding model for four representative DNA targets, including the wild-type sequence (highlighted in cyan). nd: not determined.

a DNA Target 5′-ttAAGAGAAGCAAATACGTTAta-3′
RNA aauucucaacguuuaugcaauau

RNA Target 5′-uuAAGAGAAGCAAAUACGUUAua-3′
DNA aattctcaacgtttatgcaatat

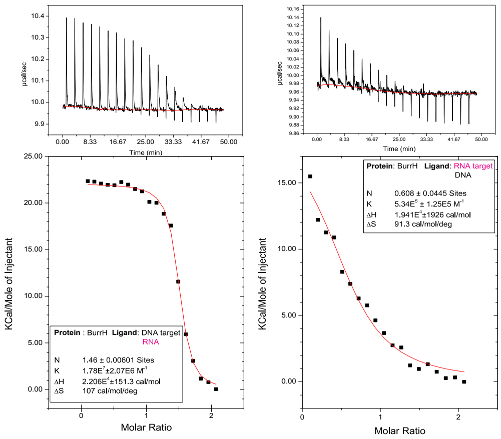

b

BurrH I I N T N I I N D I I T R S D N G G S
burrH DNA tt A A G A G A A G C A A A T A C G T T A ta
Variant1 N N HD G HD G N G N N N HD G HD N N N G HD
V1 DNA tt G G C T C T G T G G G C T C G G G T C ta
Variant2 G G N G HD HD N N N I I HD HD HD I N I N HD
V2 DNA tt T T G T C C G G G A A C C C A G A G C ta
Variant3 HD I N G N G G G I G N N G G I HD G G I
V3 DNA tt C A G T G T T T A T G G T T A C T T A ta
Variant4 N G I G I G I G G G I I N HD I HD G G I
V4 DNA tt G T A T A T A T T T A A G C A C T T A ta

c

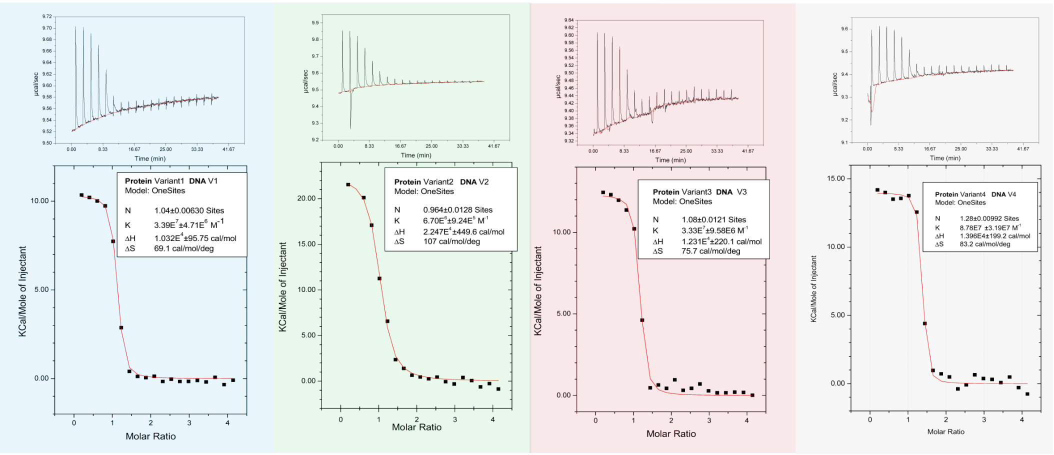

d

Protein	Ligand	ITC						
		n (sites)	K_A (M^{-1})	K_D (nM)	ΔH (kcal/mol)	TΔS (kcal/mol)	T (°C)	ΔG (kcal/mol)
BurrH	BurrH	1.1 ± 0.005	38.9 x 10^6 ± 6.6 x 10^6	25 ± 4.3	26.7 ± 0.22	37.0	25	-10.3
BurrH	DNA target/RNA	1.5 ± 0.006	17.8 x 10^6 ± 2.1 x 10^6	56 ± 6.5	22.1 ± 0.15	31.9	25	-9.8
BurrH	RNA target/DNA	0.6 ± 0.044	0.53 x 10^6 ± 0.1 x 10^6	1886 ± 438	19.4 ± 1.92	27.2	25	-7.8
Variant1	V1	1.0 ± 0.006	33.9 x 10^6 ± 4.7 x 10^6	29 ± 4.1	10.3 ± 0.09	20.6	25	-10.3
Variant2	V2	0.9 ± 0.012	6.70 x 10^6 ± 0.9 x 10^6	149 ± 20	22.5 ± 0.45	31.9	25	-9.3
Variant3	V3	1.0 ± 0.012	33.3 x 10^6 ± 9.5 x 10^6	30 ± 8.6	12.3 ± 0.22	22.5	25	-10.2
Variant4	V4	1.3 ± 0.009	87.8 x 10^6 ± 32 x 10^6	12 ± 4.1	13.9 ± 0.21	24.8	25	-10.8

Supp. Figure 4.- Thermodynamic charactrisation of DNA binding by the engineered variants in the BurrH platform. **a)** Hybrid DNA-RNA and RNA-DNA targets used for testing BurrH binding preferences, the RNA strand is colored in magenta with capital letters for the target sequence. The binding isotherms are shown below. **b)** BSRs of wild type BurrH and the engineered variants with their corresponding DNA targets (coding strand in direction 5´-3´). **c)** Raw ITC data used to fit the binding isotherms using a non-linear regression curve fitting using one site binding model. Best-fit thermodynamic parameters are reported in the insert. **d)** Table summarizing the thermodynamic parameters of the protein-DNA interaction for BurrH and the different variants. The ΔG was calculated as $-RT \ln K_A$.

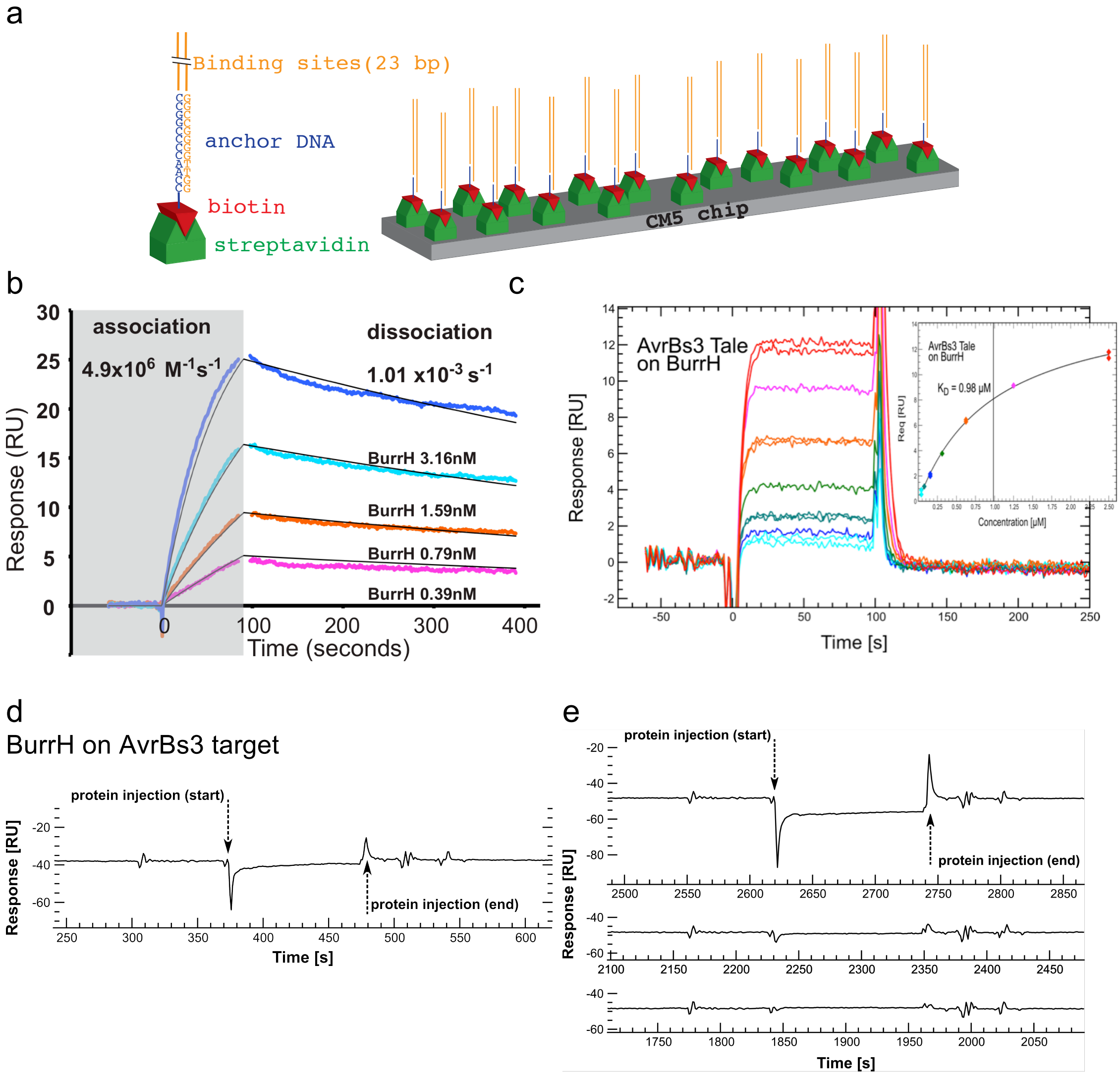

f

Protein	DNA[a]	Exp[b]	$10^{-6}k_{on}$ [M^{-1} s^{-1}]	$10^{4}k_{off}$ [s^{-1}]	K_D [nM]	Range of concentrations tested[c]
BurrH		3	2.63 ± 0.32	9.23 ± 1.43	0.36 ± 0.07	0.31 - 2.5
AvrBs3 TALE		3	9.25 ± 2.97	19.6 ± 2.3	0.22 ± 0.05	0.31 – 2.5

[a]The proteins are tested on their specific DNA targets.
[b]Number of times the interaction analysis was replicated. The reported errors are the standard deviation of the experimental replicates.
[c]Range of concentrations in nM used for the analysis. The protein samples were prepared by two-fold serial dilution from the highest concentration indicated. One or two concentrations were repeated during each interaction cycle.

Supp. Figure 5.- Binding kinetics analysis using SPR. **a)** Scheme of the experimental set up used to test BurrH-DNA binding. **b)** SPR sensorgrams of BurrH flown at increasing concentrations over its immobilized target DNA. The BuD array presents a fast association and low dissociation behaviour. **c)** TALE AvrBs3 can bind BurrH DNA target with a K_D=1µM. **d)** BurrH does not bind AvrBs3 target DNA at 1 µM concentration, showing the high specificity of this scaffold compared to TALE AvrBs3. The high concentration of BurrH was used to challenge the specificity in the worst scenario for this scaffold. Typical affinities for the specific target are in the nM range. **e)** SPR experiments testing the binding of variant 3 (at 250 nM) to the DNA targets of the other BurrH variants 1, 2 and 4. The three sensorgrams are aligned using the arrows that indicate the protein injection start and end, and no binding (SPR response) was observed. The variants 1 to 4 were tested at 250nM for binding on the BurrH DNA. In all cases, no binding was detected. The same result was obtained for the rest of the variants crossing their targets (data not shown). **f)** Table displaying the kinetic and equilibrium binding constants determined for AvrBs3 TALE and BurrH on their targets using SPR at 25°C.

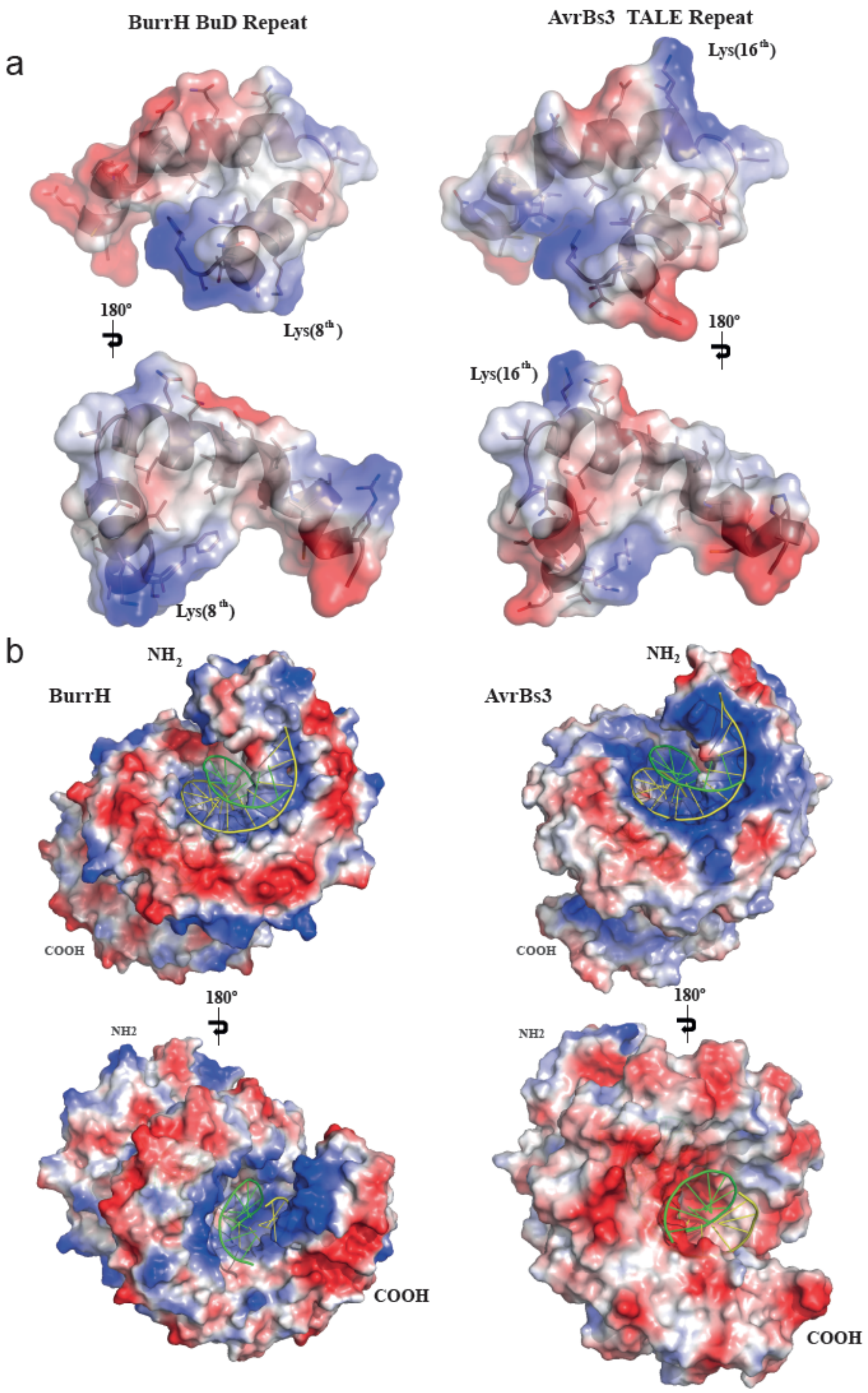

Supp. Figure 6.- Surface representation of BurrH electrostatic potential. **a)** Electrostatic surface representation of the 7th BuD repeat present in BurrH and one AvrBs3 TALE repeat showing the different charge arising from the presence of lysine in the 8th position of the BuD repeat. This residue is found in position 16th in TALE (Supp. Fig. 2). **b)** Surface representation of the electrostatic potential of BurrH and the AvrBs3 TALE. Remarkably, the electropositive (blue) and electronegative (red) potential distribution along the proteins is very different in agreement with the different repeat sequences. The coding strand is coloured in yellow and the non-coding in green.

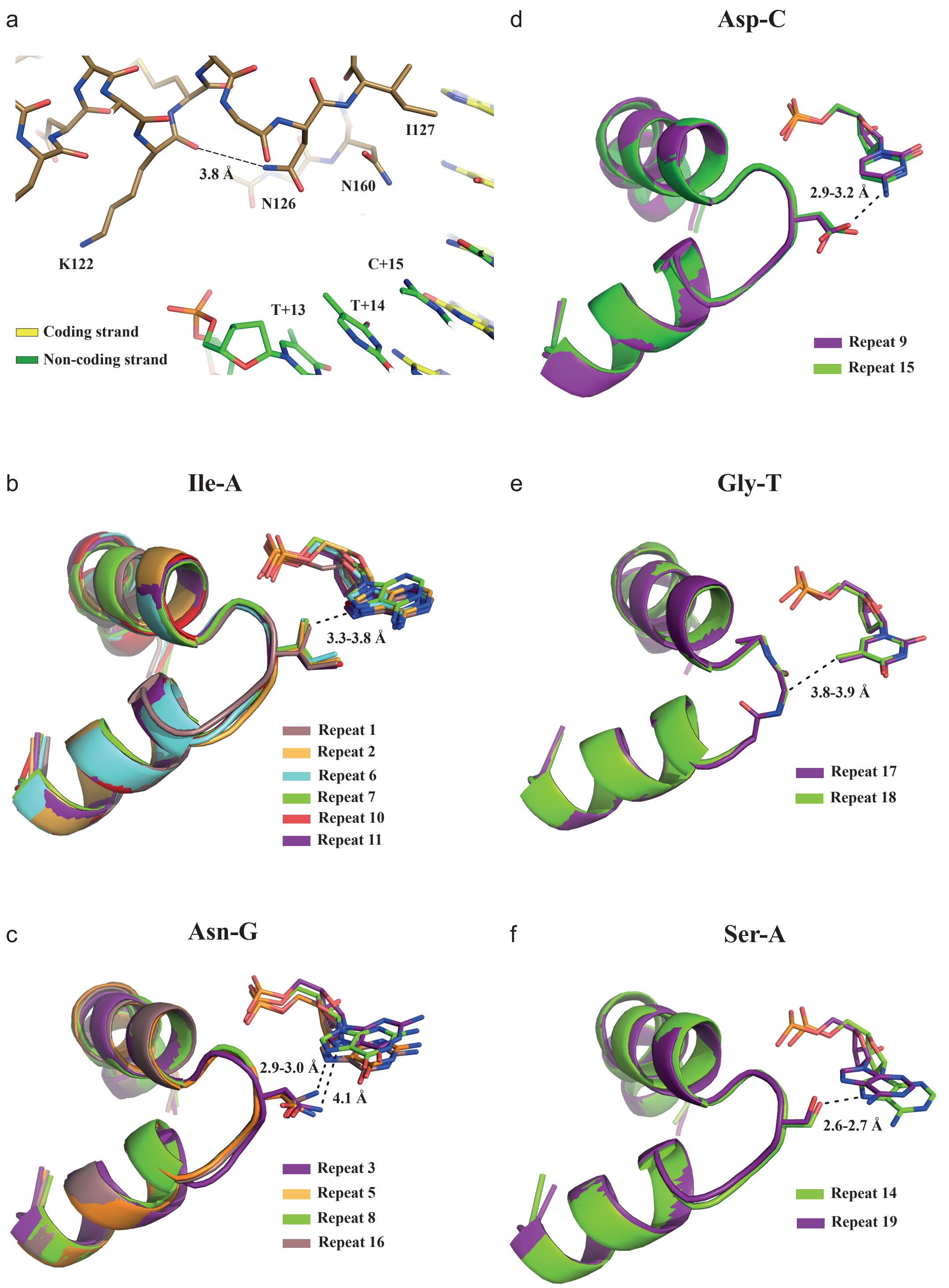

Supp. Figure 7.- Structural details of the BuD repeats. **a)** Detailed view of the interaction of the strictly conserved Asn residue in position 12th in a representative BuD repeat (see Supp. Fig 2). Contacts between the BuD BSRs (position 13th) and the DNA bases. The five combinations similar to TALEs RVDs are present in the BuD repeat BSRs and its target DNA complex as follows: **b)** The Ile-A interaction occurs through van der Waals contacts with the base. **c)** The Asn-G association involves hydrogen bonds of the amino acid side chain with the guanine ring. **d)** In the Asp-C recognition the Asp side chain interacts with the cytosine amine group. **e)** The presence of just the hydrogen allows enough space for the placement of the methyl group of the thymine in the Gly-T interaction. **f)** The hydroxyl side chain associates with the N7 imine group of adenosine in the Ser-A interaction (see Supp. Results for a detailed description).

a

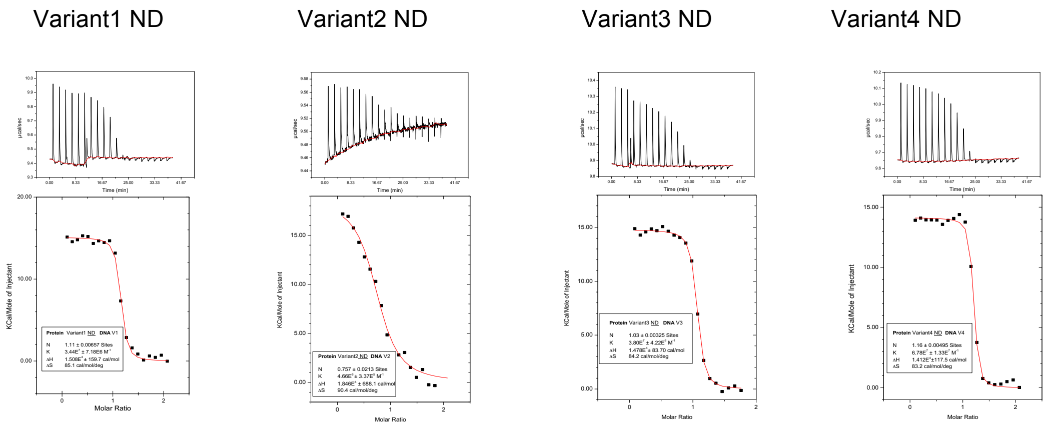

b

Protein	DNA[a]	ITC						
		n (sites)	K_A (M^{-1})	K_D (nM)	ΔH (kcal/mol)	TΔS (kcal/mol)	T (°C)	ΔG (kcal/mol)
Variant1 ND	V1	1.1 ± 0.001	$34.4 \times 10^6 \pm 7.2 \times 10^6$	29 ± 6.1	15.1 ± 0.16	25.3	25	-10.2
Variant2 ND	V2	0.7 ± 0.021	$4.66 \times 10^6 \pm 0.33 \times 10^6$	215 ± 15	18.5 ± 0.6	26.9	25	-9.06
Variant3 ND	V3	1.0 ± 0.003	$38.0 \times 10^6 \pm 0.4 \times 10^6$	26 ± 2.9	14.7 ± 0.08	25.1	25	-10.3
Variant4 ND	V4	1.2 ± 0.005	$67.8 \times 10^6 \pm 13 \times 10^6$	14 ± 2.9	14.1 ± 0.12	24.8	25	-10.6

[a]See Supp Fig 4b for the nucleotide sequences

Supp. Figure 8.- Thermodynamic properties of the single code variants 1, 3, 4 . **a)** ITC binding measurements showing that BuD arrays constructed following the single residue to nucleotide correspondence conserve the thermodynamic properties and binding affinities of the wild type BurrH. The variants BSRs and targets are identical to those in sup. Fig 4b with the exception that asparagine was used to replace the histidines in the HD dipeptides. **b)** Table containing the thermodynamic parameters of the protein-DNA interaction for the different variants. The ΔG was calculated as –RT ln K_A.

a

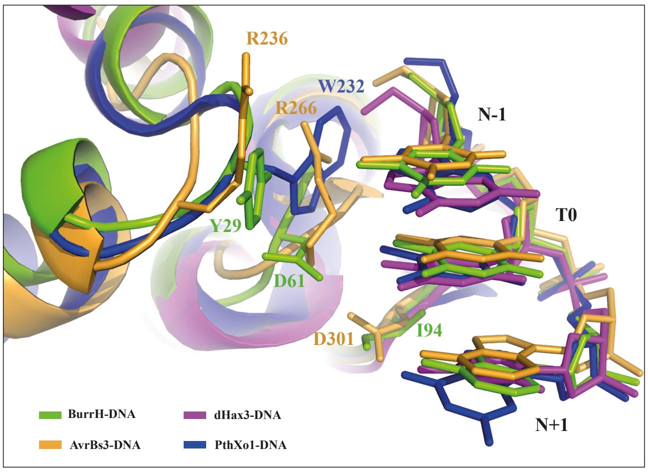

b

```
      0 1 2 3 4 5 6 7 8 9 10111213141516171819
5′- t t A A G A G A A A C A A A T A C G T T A a -3′ BurrH DNA target
      a
      c
      g
```

c

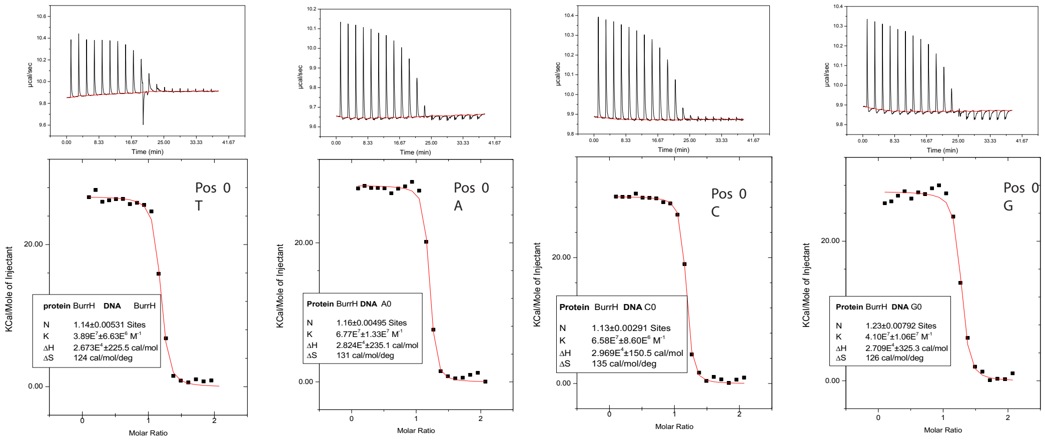

d

Protein	DNA	ITC						
		n (sites)	K_A (M^{-1})	K_D (nM)	ΔH (kcal/mol)	TΔS (kcal/mol)	T (°C)	ΔG (kcal/mol)
BurrH	BurrH	1.1 ± 0.005	38.9 x 10^6 ± 6.6 x 10^6	25 ± 4.3	26.7 ± 0.22	37.0	25	-10.3
BurrH	A0	1.1 ± 0.005	67.7 x 10^6 ± 13 x 10^6	15 ± 2.8	28.2 ± 0.24	39.8	25	-10.7
BurrH	C0	1.1 ± 0.004	65.8 x 10^6 ± 8.6 x 10^6	15 ± 3.2	29.6 ± 0.15	40.2	25	-10.6
BurrH	G0	1.2 ± 0.007	41.0 x 10^6 ± 10 x 10^6	24 ± 6.3	27.1 ± 0.32	37.5	25	-10.3

Supp. Figure 9.- N-terminal region and base preference at the 0 position of the target. **a)** Structural comparison of the T0 region between TALEs and BurrH. **b)** Sequences of the DNA targets used in the ITC measurements of BurrH binding its DNA target containing one of the possible nucleotides at position 0 (T, A, C or G). **c)** ITC raw data (upper panels) with the corresponding binding isotherms and non-linear regression curve fitting to a one site binding model (lower-panels). The thermodynamic parameters for each experiment are reported in the inserts. **d)** Table summarizing the thermodynamic parameters of the protein-DNA interaction of BurrH with the four different targets. The ΔG was calculated as –RT ln $K_{a.}$

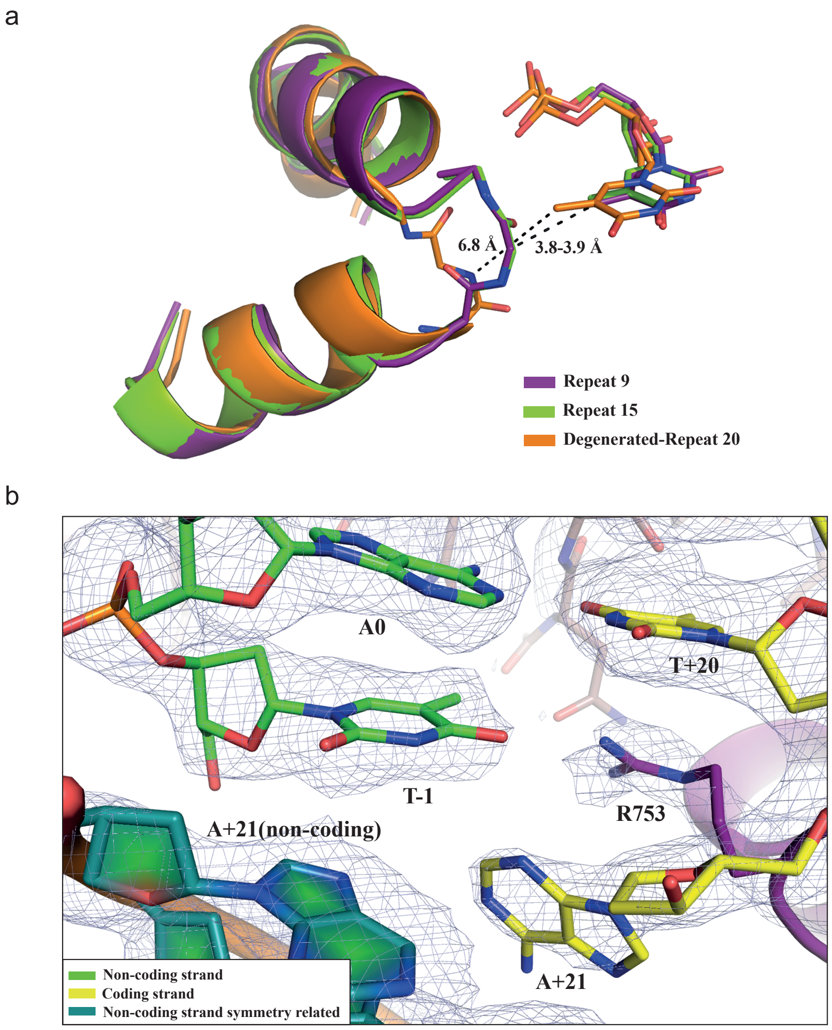

Supp. Figure 10.- Structural details of the C-terminal region. **a)** Comparison of the interaction between Gly721 and T_{+20} in the 20th degenerated repeat compared with the Gly-T interaction found in the 9th and 15th BuD repeat. **b)** View of the Arg753 interactions disrupting the final base pair (A_{+21}-T_{-1}) in the 21st degenerated repeat.

a b

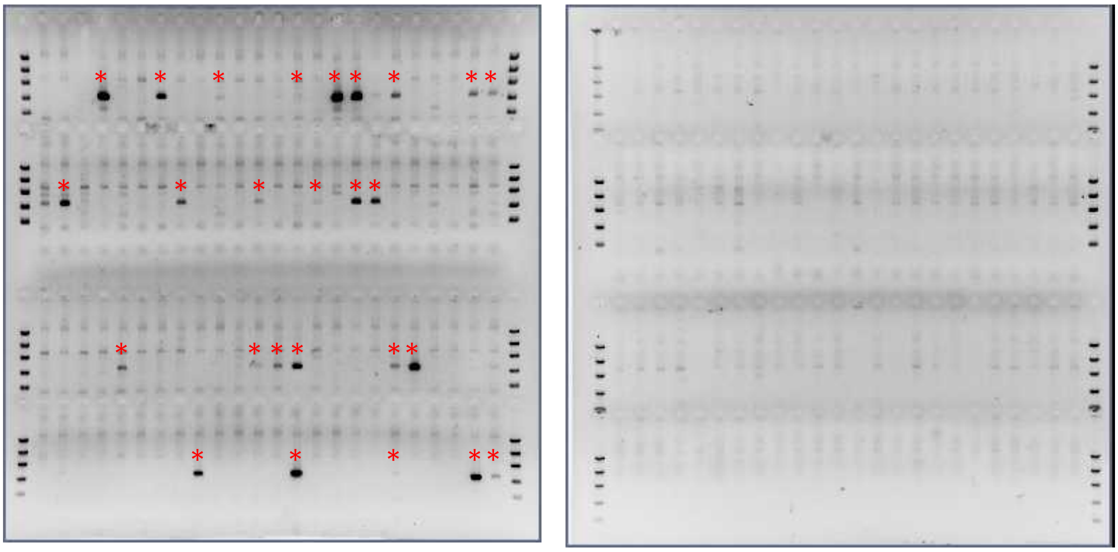

c

	10 cells/well analysis				
	Number of analyzed wells	Wells PCR +	Transfection efficacy	Estimated plating efficacy	**TGI Frequency**
BuDsN1/N2 + donor DNA	288	93	38%	30%	**25.5%**
donor DNA only	96	0	57%	30%	**0%**

Supp. Figure 11.- Targeted gene insertion (TGI) frequency was calculated as in Daboussi, F. et al. *Nucleic Acids Res* **40**, 6367-79, (2012). **a)** TGI events PCR screening of 96 well at the HBB locus in the presence the BuD-based nuclease (BuDN1/N2 HBB) and donor DNA (positive wells are indicated with a red star). **b)** Same as for (a) but with the donor DNA only. **c)** Targeted Gene Insertion frequency determined at the HBB locus in the presence or absence of the BuD-based nucleases (BuDN1/N2 HBB), taking in account the transfection and plating efficacy. The results are plotted in Fig 5e.
